# Quantitative Impact of Cell Membrane Fluorescence Labeling on Phagocytosis Measurements in Confrontation Assays

**DOI:** 10.3389/fmicb.2020.01193

**Published:** 2020-06-05

**Authors:** Zoltan Cseresnyes, Mohamed I. Abdelwahab Hassan, Hans-Martin Dahse, Kerstin Voigt, Marc Thilo Figge

**Affiliations:** ^1^Applied Systems Biology, Leibniz Institute for Natural Product Research and Infection Biology – Hans Knöll Institute, Jena, Germany; ^2^Jena Microbial Resource Collection, Leibniz Institute for Natural Product Research and Infection Biology – Hans Knöll Institute, Jena, Germany; ^3^Institute of Microbiology, Faculty of Biological Sciences, Friedrich Schiller University Jena, Jena, Germany; ^4^Department of Pests and Plant Protection, National Research Centre, Giza, Egypt; ^5^Infection Biology, Leibniz Institute for Natural Product Research and Infection Biology – Hans Knöll Institute, Jena, Germany

**Keywords:** confrontation assay, automated image analysis, host–pathogen interaction, phagocytosis, label-free imaging, spinning disk confocal microscopy

## Abstract

Phagocytosis is series of steps where the pathogens and the immune cells interact during an invasion. This starts with the adhesion process between the host and pathogen cells, and is followed by the engulfment of the pathogens. Many analytical methods that are applied to characterize phagocytosis based on imaging the host–pathogen confrontation assays rely on the fluorescence labeling of cells. However, the potential effect of the membrane labeling on the quantitative results of the confrontation assays has not been studied in detail. In this study, we determine whether the fluorescence labeling processes themselves influence the results of the phagocytosis measurements. Here, alveolar macrophages, which form one of the most important compartments of the innate immune system, were used as an example of host cells, whereas *Aspergillus fumigatus* and *Lichtheimia corymbifera* that cause aspergillosis and mucormycosis, respectively, were studied as examples for pathogens. At first, our study investigated the importance of the sequence of steps of the fixation process when preparing the confrontation assay sample for microscopy studies. Here we showed that applying the fixation agent before the counter-staining causes miscalculations during the determination of the phagocytic measures. Furthermore, we also found that staining the macrophages with various concentrations of DID, as a typical membrane label, in most cases altered the capability of macrophages to phagocytose FITC-stained *A. fumigatus* and *L. corymbifera* spores in comparison with unlabeled macrophages. This effect of the DID staining showed a differential character dependent upon the labeling status and the specific type of pathogen. Moreover, labeling the spores of *A. fumigatus* and *L. corymbifera* with FITC increased the phagocytic measures during confrontation with unlabeled macrophages when compared to label-free spores. Overall, our study confirms that the staining process itself may significantly manipulate the quantitative outcome of the confrontation assay. As a result of our study, we also developed a user-friendly image analysis tool that analyses confrontation assays both with and without fluorescence labeling of the host cells and of the pathogens. Our image analysis algorithm saves experimental work effort and time, provides more precise results when calculating the phagocytic measures, and delivers a convenient analysis tool for the biologists to monitor host–pathogen interactions as they happen without the artifacts that fluorescence labeling imposes on biological interactions.

## Introduction

Microorganisms, including bacteria, fungi, viruses, and parasites, can cause severe infectious diseases to living creatures including humans, and rank among the top ten causes of death such as lower respiratory, diarrheal, and tuberculosis diseases according to the recent report of World Health Organization (WHO^1^). About one quarter of the human population worldwide suffers from skin diseases that are caused by fungi, including one and a half million death cases that were reported to be caused by invasive fungal diseases (Brown et al., 2012). The immune system has several ways to combat the invasion of opportunistic fungi and to reduce their damage (Sperandio et al., 2015). The innate immune system consists of several immune cells such as monocytes/macrophages, endothelial cells, granulocytes, natural killer cells (NK), and dendritic cells (DCs) that constitute the first line of defense against the foreign particles and invading microorganisms (Gasteiger et al., 2017). Various immune cells are professional phagocytes, such as macrophages, neutrophils, and dendritic cells that have a key role in recognizing and killing the pathogens. Phagocytosis is one of the most important responses of immune cells against invading pathogens and particles that depends solely on the recognition step that occurred through adhesion, an event that was first observed by Élie Metchnikoff in 1880 (Rosales and Uribe-Querol, 2017). The first steps in the phagocytosis process are the adhesion and recognition that occur through receptors that are present on the surface of the plasma membrane of immune cells, known as pattern recognition receptors (PRRs) (Erwig and Gow, 2016; Rosales and Uribe-Querol, 2017). PRRs could recognize specific components that are found on the surface of fungal cells known as pathogen associated molecular patterns (PAMPs) (Erwig and Gow, 2016). Collectins form a class of PRRs that belong to C-type lectin-like domains that have a key role in enhancing the immune interaction in the lung against pathogens. Such roles include the acceleration of the killing process of pathogens, the increasing of the rate of their phagocytosis by macrophage cells, and the control of the inflammation process. Collectins have various candidates of surfactant proteins such as SP-A (surfactant-protein A), which is important in orchestrating the function of macrophage cells (Casals et al., 2018). Due to the importance and essence of the phagocytosis process in understanding the immune response toward pathogenic fungi, several studies showed that each immune cell has its own phagocytosis ratio to each fungal species and even it can be strain-specific in the same fungal species due to a difference in their surface structure or a genetic modification (Kraibooj et al., 2015; Hassan et al., 2019).

Various studies were conducted to provide the optimal protocols to measure the phagocytosis ratio of several fungal species by various immune cells relying on automated image analysis of microscopy studies (Mech et al., 2011, 2014; Kraibooj et al., 2014, 2015; Mattern et al., 2015; Hassan et al., 2019). Monitoring the phagocytosis process of opportunistic pathogens by immune cells is a matter of interest where image-based techniques appeared amongst the most useful, standardized, and progressively developed methods during the last decade (Medyukhina et al., 2015). These methods proved their efficacy in reducing the manual effort, being objective and more precise, as well as being capable to analyze very large numbers of immune cells and pathogens during phagocytosis processes compared to either manual counting or flow cytometry analysis (Zhou and Wong, 2006; Hassan et al., 2019). Importantly, flow cytometry allows for enumerating cells that are associated with each other, but does not distinguish between pathogens that have been phagocytozed by immune cells or that just adhere to the phagocyte. At the same time, microscopy techniques can overcome this problem as we showed in the current study and previous work (Hassan et al., 2019). Moreover, flow cytometry methods rely on fluorescence staining methods that have a direct influence on the outcome of the phagocytosis assays, as shown by the current study. However, the microscopy-based method presented in this work can calculate all phagocytic measures precisely with only one counter-staining being necessary. Microscopy analysis also characterizes the morphological characters of the pathogens and host cells, whereas flow cytometry does not provide such morphokinetic characterization. At the same time, microscopy techniques produce a large enough number of events to provide statistical validation for a much wider range of parameters such as solidity, perimeter, area, roughness, etc., which cannot be achieved through flow cytometry analysis. As an alternative, imaging flow cytometry could be used with some success. Here the main problem lies with the reduced spatial resolution, which will make it difficult to apply label-free segmentation methods.

In our previous studies, an automated image analysis technique was established by a customized Algorithm for Confrontation Assay Quantification (ACAQ), which was initially based on the commercial Cognition Network Technology of the Definiens Developer XD platform (Mech et al., 2011, 2014; Kraibooj et al., 2014) (ACAQ-v1). This version of ACAQ could not successfully segment the host cells individually; thus, we added new features to our algorithm (ACAQ-v2), which enabled segmentation of the host cells and subsequently provided the values of phagocytic measures such as the symmetrized phagocytic index based on distinguishing fluorescently labeled phagocytes, free spores, adherent spores, and phagocytosed spores (Kraibooj et al., 2015). In our more recent study, the segmentation was further advanced by recognizing phagocytes without staining relying on Hessian filters (Cseresnyes et al., 2018; Hassan et al., 2019) (ACAQ-v3). In this study, we made the next logical step and extended the features of ACAQ-v3 to count and measure spores without fluorescence labeling as well. These algorithmic improvements will have several advantages in various biological applications such as (i) saving labor efforts, (ii) reducing lab consumables, and (iii) avoiding staining artifacts that may affect the phagocytosis process and manipulate its quantification. Moreover, automated image analysis provides all details about the spatial aspects of host–pathogen interactions compared to FACS analysis as discussed before (Hartung et al., 2019; Hassan et al., 2019).

Previous studies relied heavily on staining methods for either the spores of fungi, or the immune cell, or both. In our current study, we evaluate whether chemical substances that were applied for staining the cell membranes manipulate the value of phagocytosis ratio during host–pathogen interactions. Murine alveolar macrophages (MH-S cells) are chosen as an example for immune cells because of their biological function belonging to the first line of defense of innate immunity against inhalation of airborne fungal spores, which is considered as the primary route of infection (Martin and Frevert, 2005). Moreover, about 95% of the leukocytes in the murine lung consist of MH-S cells (Martin and Frevert, 2005). Furthermore, MH-S cells secrete several signaling compounds that recruit other immune cells to the site of infection (Martin and Frevert, 2005). From the point of view of pathogens, one strain of *Aspergillus fumigatus* is included in the current study as an example of the main causative agent of aspergillosis that is considered to be the main cause of disease in immunocompromised patients (Webb et al., 2018), as well as two strains of *Lichtheimia corymbifera* as causative agents of mucormycosis that is a life-threatening disease in patients suffering from immune system deficiency with a rate of mortality that is tremendously increasing worldwide during the last decade (Hassan and Voigt, 2019).

Fluorescein isothiocyanate (FITC) is a useful reagent for labeling spore proteins through binding to α-amino groups of the N-terminal amino acids at pH < 9.5. However, non-covalent dye-binding was observed during interaction with some proteins such as lysozyme and albumin (Kawauchi et al., 1969). Besides its several applications in labeling the lectins and antibodies, FITC is also used for determination of intracellular and intra-organelle pH (Lanz et al., 1997). To our best knowledge, studies about the influence of FITC staining on the phagocytosis ratio of opportunistic fungi by professional immune cells do not exist. In our study, the effect of FITC staining on the capability of MH-S cells to engulf the spores of two strains of *L. corymbifera* and one strain of *A. fumigatus* is studied thoroughly. DID [DiIC18 (5); 1, 1′-dioctadecyl-3, 3, 3′, 3′ tetramethylindodicarbocyanine, 4-chlorobenzenesulfonate salt] is a lipophilic dye that is widely used to determine the proliferation of tumor, phagocytosis and cell transplantation *in vivo* (Kraibooj et al., 2014; Yumoto et al., 2014; Zarychta-Wiśniewska et al., 2017). Even though there are several studies measuring the effect of DID on cell differentiation, apoptosis, proliferation, production of reactive oxygen species (ROS) (Mohtasebi et al., 2014), there is no study concerning whether DID staining of professional immune phagocytes has side effects on the value of the phagocytosis ratio of opportunistic fungi. In the current study, we determined the role of DID staining in phagocytosis ratio by MH-S cells. Moreover, the importance of the sequence of fixation steps after finalizing the phagocytosis process is tested. To our best knowledge, this is the first study dealing in detail with the role of host and pathogen staining in the phagocytosis process of pathogens by professional immune phagocytes. Furthermore, the current study has an interdisciplinary character that contributes to the improvement of the automated bioimage processing from a systems biology point of view and gives clear evidence about the role of staining techniques, which are applied daily in most biological labs to monitor cell–cell interactions.

## Materials and Methods

### Growth Conditions of Fungal Strains

Two strains of *L. corymbifera* JMRC:FSU:09682, JMRC:FSU:10164, and *A. fumigatus* wild-type ATCC 46645 are included in our study. *Lichtheimia* strains were cultivated on Malt extract medium (40 g of malt extract, 15 g of agar, and 4 g of yeast extract per one liter) at 37°C for 7 days. *Aspergillus* strain was cultivated on *Aspergillus* minimal medium (AMM) [10 gl^–1^ glucose, 7 mM KCL, 2.1 mM MgSO_4_, 11.2 mM KH_2_PO_4_, 70 mM NaNO_3_, 15 gl^–1^ agar, and 1 μl/ml trace element solution] for 5 days at 37°C. The plates were washed with Phosphate buffered saline (PBS) solution to collect the spores (Hassan et al., 2019). The components of PBS were as follows: 8 gl^–1^ NaCl, 0.2 gl^–1^ KCl, 0.2 gl^–l^ K_2_HPO_4_, and 1.44 gl^–l^ Na_2_HPO_4_ at pH 7.4. All chemicals that were involved in this reagent were purchased from Carl Roth, Germany.

Spores used in our study were either unlabeled or labeled by 0.1 mg/ml FITC (Fluorescein isothiocyanate, Sigma Aldrich Chemie GmbH, F3651) dissolved in PBS (0.1 mg/ml) in 0.1 M Na_2_CO_3_ for 30 min at 30°C. The FITC-stained spores were then washed three times with PBS in order to remove the excess FITC. The number of spores was determined by Thoma chamber and re-suspended in RPMI-1640 culture medium (BE12-16F; Lonza, Verviers, Belgium) (Hassan et al., 2019).

### Growth Conditions of Murine Alveolar Macrophages

Murine alveolar macrophages (MH-S) (ATCC CRL-2019^TM^) were used in our study for phagocytosis process. They were cultivated on RPMI-1640 containing 10% (v/v) heat inactivated bovine serum (ATCC-30-2020), 1% (w/v) sodium bicarbonate (Lonza, Köln, Germany), (50 mg ml^–1^) gentamycin sulfate (17-518Z;Lonza), and 0.05 mM beta-mercaptoethanol (Life Technologies, Darmstadt, Germany) in a humidified 5% CO_2_ incubator at 37°C. In our study, the unlabeled and labeled MH-S cells were involved in our study. For labeling MH-S cells, Vybrant DID (Life Technology GmbH, Darmstadt, Germany) was applied with various concentrations (1, 2, 3, 4, and 5 μl/ml, according to the guidelines provided by the manufacturer) to MH-S cells for 15 min and MH-S cells were washed two times with RPMI medium to remove the excess of DID dye directly before carrying out the experiments. In some experiments, MH-S cells were labeled with antibody after the phagocytosis process as follows: a monoclonal rat anti-CD9 antibody (1:200; Santa Cruz Biotechnology, Heidelberg, Germany) was applied overnight at 4°C and then cells were incubated for 1.5 h with an Alexa Fluor 647 Goat Anti-Rat IgG antibody (1:200; Life Technologies) at room temperature. These cells were utilized in experiments where the intracellular localization of labeled spores was studied in order to quantify the erroneous counting of adherent cells that were in fact inside the MH-S cells.

### Confrontation Assay

2^∗^10^5^ cells of MH-S cells were seeded per well on glass coverslips in 24-well plates (NUNC; 142475) and let adhere overnight. FITC-labeled or unlabeled spores were added at multiplicity of infection (MOI) 5 (five spores for each macrophage). A centrifugation step for 5 min at 100 g was applied to synchronize the spores and MH-S cells, as recommended by a recent publication (Ninkoviæ and Roy, 2014). The plate was incubated at 37°C in a humidified 5% CO_2_ incubator for 1 h to initiate the phagocytosis process. To stop the phagocytosis process, the media was removed and ice-cold PBS was added twice to remove the excess of spores. To discriminate between the engulfed spores and extracellular spores, each well was incubated with 500 μl of calcofluor white solution (CFW; Sigma Aldrich Chemie GmbH, F3543) (0.5 mg ml^–1^ CFW in PBS) for 15 min at room temperature. CFW was discarded and PBS solution was applied twice to remove the excess of CFW staining. The cells were fixed for 15 min at room temperature with 3.7% (vol/vol) formaldehyde in PBS. The formaldehyde-containing PBS was prepared from a stock solution of 37% (vol/vol) formaldehyde (Roth, 4979) containing 9–14% methanol. Consequently, the final concentration of methanol was 0.9–1.4%. An amount of 500 μl of this solution was added to each well in the plate. The cells were thus exposed to a low concentration of methanol (0.9–1.4% vol/vol) that would be unlikely to affect the membrane structure. Cells were washed two times again with PBS. Roti^®^-mount fluorcare (ROTH) was implemented as anti-fading agent. Three biological replicates were carried out for each experiment in our current study for statistical analysis.

### The Effect of the Fixation Process on the Outcome of the Confrontation Assay

In order to determine whether the sequence of CFW staining and formaldehyde fixation influences the value of the phagocytosis measurement, two types of experiments were carried out. In one set of experiments, CFW was applied first, followed by 3.7% (vol/vol) formaldehyde fixation. In the other set of experiments, the order was reversed, i.e., first 3.7% (vol/vol) formaldehyde was used, followed by CFW staining.

### Image Acquisition

The samples were imaged with a Zeiss Axio Observer 7 Spinning Disk Confocal Microscope (SDCM, Carl ZEISS Jena, Germany) and images were acquired with the ZEN 2.1 software (ZEISS) using a 63x NA1.4 objective lens. A minimum of 7 tile scans were scanned for each sample in each replicate. In Supplementary Figure S1, representative examples from *L. corymbifera* strain JMRC: FSU: 10164 are shown to illustrate the labeling and imaging sequence. Images were acquired using transmitted light in bright field mode to capture both the MH-S cells and the spores (Supplementary Figures S1A–C, gray), together with the green fluorescence of FITC (labeling all spores; Supplementary Figures S1A–D, green) and the blue fluorescence of CFW that served as a counter-stain to label only the non-phagocytosed spores (Supplementary Figures S1A–C,E, blue). The clear difference between the phagocytosed spores being only green and the non-phagocytosed spores being both green and blue (Supplementary Figure S1F) indicate that CFW can indeed be used as approximate guidance to distinguish between engulfed and non-engulfed spores. In order to check our label-free cell segmentation algorithm, in a series of experiments MH-S cells were labeled with a monoclonal rat anti-CD9 antibody as described above (Supplementary Figures S1G–I, red color showing the labeled MH-S cell membrane).

### Image Processing

The automated image processing was carried out as we previously described (Kraibooj et al., 2015; Cseresnyes et al., 2018). Briefly, confocal images were processed by custom-written software inside the Fiji framework (Schindelin et al., 2012). Images of fluorescence and transmitted light illumination were used to segment the MH-S cells, as well as free, adherent and phagocytosed spores (Supplementary Figure S2). The phagocytic measures were calculated based on the following four equations:

Phagocytosis ratio:

(1) $$\Phi_{\text{p}}=\frac{N_{\text{p}}^{\text{phag}}}{N_{\text{p}}^{\text{phag}}+N_{\text{p}}^{\text{adh}}},$$

Uptake ratio:

(2) $$\Phi_{\text{h}}=\frac{N_{\text{h}}^{\text{phag}}}{N_{\text{h}}},$$

Phagocytic index:

(3) $$\Phi_{\text{i}}=\frac{N_{\text{p}}^{\text{phag}}}{N_{\text{h}}}\cdot \Phi_{\text{h}},$$

Symmetrized phagocytic index:

(4) $$\Phi_{\text{i}}^{\text{sym}}=\Phi_{\text{p}}\cdot \Phi_{\text{h}}.$$

In Eqs. (1) to (4), we used the following definitions:

$N_{\text{p}}^{\text{phag}}$ The number of phagocytosed (FITC positive, CFW negative) pathogens.
$N_{\text{p}}^{\text{adh}}$ The number of adherent (FITC positive, CFW positive) pathogens.
$N_{\text{h}}^{\text{phag}}$ The number of phagocytosing host cells.
*N*_*h*_ The total number of segmented macrophages.

The phagocytosis ratio Eq. (1) measures the proportion of phagocytosed pathogens relative to the total number of associated spores. Eq. (2) describes a similar measure from the view point of the host cells, i.e., the ratio of those host cells amongst all host cells in the image that have phagocytosed at least one pathogen. The phagocytic index in Eq. (3) provides the per-image average number of the phagocytosed pathogens per host cell, while the symmetrized phagocytic index Eq. (4) indicates the overall probability of any pathogen to be phagocytosed by a host cell. The symmetrized phagocytic index is the combination between the uptake ratio and the phagocytic index and is chosen to be used in the current study as the most comprehensive measurement for phagocytosis processes (Cseresnyes et al., 2018).

In addition, the previously introduced analysis tool ACAQ-v3 (Cseresnyes et al., 2018) was further developed to provide a label-free segmentation and quantification method for both the host cells and the pathogens. The improved version of the analysis software (ACAQ-v4) was able to detect the spores of various species of microorganisms, e.g., *Mucor circinelloides*, *Lichtheimia corymbifera*, *Lichtheimia ramosa*, *Aspergillus fumigatus*, and the yeast cells of *Saccharomyces cerevisiae* (Supplementary Figure S3). In addition to the MH-S cells, ACAQ-v4 was also able to segment bone marrow-derived macrophage (BMDM) cells in confocal transmitted light images (Supplementary Figure S3). We present the results of such analysis by providing the morphological measures of the area, aspect ratio and solidity for all the above mentioned cell types (Supplementary Figure S3). The various cells are well separable based on their morphometric characteristics as shown in both panel A and B in Supplementary Figures S3A,B. Thus, ACAQ-v4 provides an analysis tool that can be applied to test the biological measures of a wide range of cells both with and without relying on fluorescence labeling.

In all experiments, a total of more than a hundred thousand MH-S cells and more than 170 thousand spores were counted using our analysis tool. For detailed numbers (see Table 1).

**TABLE 1:** The number of segmented MH-S cells and fungal spores per condition used in this study.

Applied labeling DID/FITC	Number of MH-S cells *N*_h_			Number of spores *N*_p_		
		
	*L. corymbifera* JMRC:FSU:09682	*L. corymbifera* JMRC:FSU:10164	*A. fumigatus* ATCC 46645	*L. corymbifera* JMRC:FSU:09682	*L. corymbifera* JMRC:FSU:10164	*A. fumigatus* ATCC 46645
Yes/yes	9928	11160	12643	23315	24282	28843
No/yes	5461	5029	2934	11439	11997	5679
Yes/no	14899	14976	18129	10864	12300	36988
No/no	1851	939	3976	1381	748	7376

*Each number is a sum of three independent biological replicates, collected for all DID concentrations. The total number of segmented MH-S cells was 101925, whereas the total number of segmented spores added up to 175212*.

### Statistical Analysis

Three independent biological replicates were performed for each treatment. Paired *t*-test was applied for determination the significance. The significance was calculated through *P*-value: “ns” means there is no significant difference as *p* > 0.05, “^∗^” means it is significant as *p* < 0.05, “^∗∗^” *p* < 0.01, and “^∗∗∗^” *p* < 0.001.

## Results

### Effort and Costs of Cell Labeling Compensated by Improved Computational Image Analysis

**FIGURE 1:**
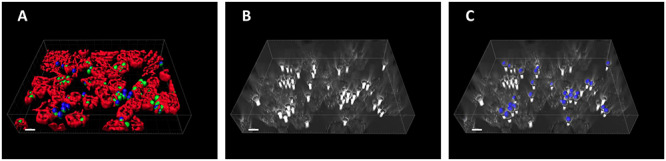
Example images showing fluorescently labeled as well as unlabeled macrophages and fungal spores as they appear during a confrontation assay. **(A)** MH-S cells were labeled with a monoclonal rat anti-CD9 antibody (red; for technical details, see section “Materials and Methods”), whereas spores of the attenuated *Lichtheimia corymbifera* strain JMRC: FSU: 10164 were labeled with FITC (green). The antibody labeling assured that the MH-S cell membranes could be precisely reconstructed in 3D, so that we could count the spores that were located inside these cells. Moreover, antibody labeling did not affect the phagocytosis process (data not shown). **(B)** MH-S cells and the spores of an attenuated *L. corymbifera* strain JMRC: FSU: 10164 were imaged using only transmitted light bright field (TL-BF) microscopy. **(C)** The final identification of the non-phagocytosed spores was achieved by counter-staining with CFW (blue color).

Traditionally, cells in confrontation assays are fluorescently labeled in order to be identified by image analysis tools. Such fluorescence-based algorithms are simple, but the experiments are associated with a substantial effort and the financial burden for the staining procedure. In addition, the labeling process and the fluorescent markers themselves may interfere with the biological process of phagocytosis. Alternatively, the image analysis process should be further developed in the direction of being able to analyze label-free cells. In our current study, we proceeded along this route and modified our ACAQ-v3 tool so that it is able to segment label-free fungal spores as well. The advanced algorithm is based on Hessian filtering, similarly to ACAQ-v3, but extended by pre- and post-processing steps as required for the segmentation of label-free spores. At the end of the phagocytosis process, it is still required to make one counter-staining step in order to distinguish those spores that remained outside of the MH-S cells (adherent and non-associated spores) from the unlabeled spores that were taken up by the MH-S cells (phagocytosed spores). In Figure 1, we illustrate this by representative images for labeled and label-free scenarios. In Figure 1A the DID-labeled MH-S cells (red color) and the FITC-labeled spores (green color) with subsequent CFW-labeling (blue color) are shown as imaged in fluorescence mode by a confocal microscope, whereas Figure 1B shows the same sample imaged in transmitted light mode. Comparing Figures 1A,B, it is evident that the difficulty of segmenting the unlabeled objects is in the absence of proper contrasts in the pixel-intensities of images. The advantage of the Hessian filter lies in the detection of morphological properties of the regions of interest (14). For properly chosen radii this filter assures that the higher-curvature objects (i.e., the spores) appear brighter than the lower curvature MH-S cells with larger size. However, since adherent and phagocytosed spores have the same morphology, one counter-staining step is still necessary, which is typically done with CFW after cell fixation (Figure 1C, blue spores). Our improvement of the ACAQ-v3 algorithm to also include the label-free segmentation of the spores thus provides a valuable tool for confrontation assay analysis with just one counter-staining (Figure 1C).

### The Sequence of Cell Fixation Steps May Obscure Phagocytosis Measurements

In previous studies, the confrontation process between FITC-labeled spores and DID-labeled MH-S cells were stopped to stain the non-phagocytosed spores with CFW in order to distinguish them from the engulfed spores. As all spores (phagocytosed or non-phagocytosed) initially carry green color from the FITC-staining, the blue CFW-stain serves as the counter-stain. This differential staining approach assumes that CFW remains extracellular and does not penetrate MH-S cells. We observed, however, that stopping the ongoing host–pathogen interactions in the confrontation assay by cell fixation with formaldehyde and subsequent CFW-staining leads to a largely increased number of CFW-positive engulfed spores, even though as phagocytosed spores they are expected to only be stained with FITC. In light of the membrane altering effect of formaldehyde (Thavarajah et al., 2012), the erroneous entry of CFW into the MH-S cells and the consequent artificial staining of the already phagocytosed spores may not appear unexpected. However, our systematic study reveals potentially important details of this artifact and allows the quantification of the error caused by this order of the labeling process.

**FIGURE 2:**
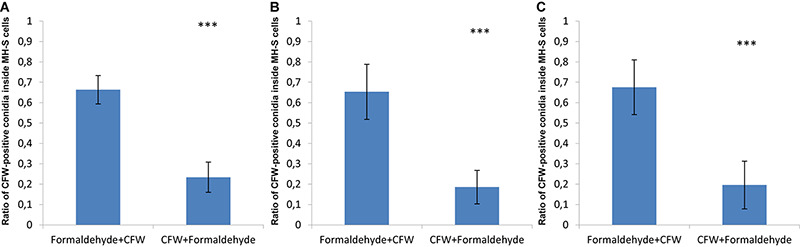
The order of fixation steps affects the accuracy of phagocytic parameter measurements of various spores confronted by MH-S cells. MH-S cells were labeled with a monoclonal rat anti-CD9 antibody (for details, see section “Materials and Methods”), whereas the spores were labeled with FITC and counter-stained with CFW. See Supplementary Figures S1–S4 for more details and for the 3D visualization of the labeled samples. **(A)** *Aspergillus fumigatus* strain ATCC 46645, **(B)** *L. corymbifera* JMRC:FSU:10164, and **(C)** *L. corymbifera* JMRC:FSU:09682. Three independent biological replicates were carried out. Statistical difference was described as: “ns” means there is no significant difference, ^∗^*p* < 0.05, ^∗∗^*p* < 0.01, ^∗∗∗^*p* < 0.001. The data represent mean ± standard deviation.

In order to visualize this phenomenon, confrontation assays were imaged by confocal microscopy in three spatial dimensions (3D) after the fixation and staining procedures were performed; either starting with formaldehyde fixation followed by CFW-staining or the other way around. The surface reconstructions of such image stacks are shown in Supplementary Figures S4–S6 and in Supplementary Videos S1–S6 as animated 3D reconstructions for the three fungal pathogens. The observed differences resulting from the order in formaldehyde fixation and CFW-staining are quantified in Figure 2. About 70% of phagocytosed spores of *A. fumigatus* were CFW-positive, i.e., they showed the blue staining despite being inside MH-S cells (Figure 2A). Similarly, about 60% of the engulfed spores of the virulent and attenuated strains of *L. corymbifera* were stained with blue color as a consequence of this fixation order (Figures 2B,C).

Applying formaldehyde fixation before counter-staining by CFW was initially done to stop the confrontation assay, i.e., to avoid ongoing phagocytosis that would possibly take place after CFW-staining, if this was applied before the cell fixation. However, applying CFW-staining first followed by formaldehyde fixation showed that the percentage of engulfed spores with CFW-positive stain in MH-S cells did on average not exceed 20% for spores of *A. fumigatus*, as well as the two strains of *L. corymbifera* (Figure 2). This suggests that applying CFW-staining first affects the quantification of phagocytosis much less than performing the fixation first. Moreover, these findings motivated us to improve the computational image analysis, because – apart from the experimental effort and the financial burden – the phagocytosis measurements may be obscured by the cell staining itself.

### DID-Stained MH-S Cells Have Concentration-Dependent Impact on Phagocytosis of Fungal Spores

**FIGURE 3:**
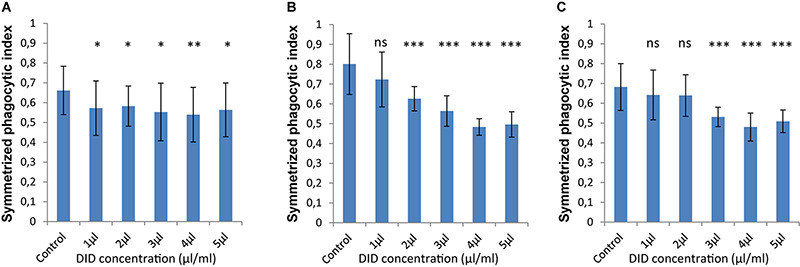
DID staining of MH-S cells alters the symmetrized phagocytic index values for FITC-labeled spores of *L. corymbifera* and *A. fumigatus* during confrontation assays with MH-S cells. **(A)** Symmetrized phagocytic index for *A. fumigatus* strain ATCC. **(B)** Symmetrized phagocytic index for the attenuated *L. corymbifera* strain JMRC: FSU: 10164. **(C)** Symmetrized phagocytic index for the virulent *L. corymbifera* strain JMRC: FSU: 09682. Three independent biological replicates were carried out. Statistical difference was always measured in comparison to the control experiment and was described as: “ns” means there is no significant difference, ^∗^*p* < 0.05, ^∗∗^*p* < 0.01, ^∗∗∗^*p* < 0.001. The data represent mean ± standard deviation.

**FIGURE 4:**
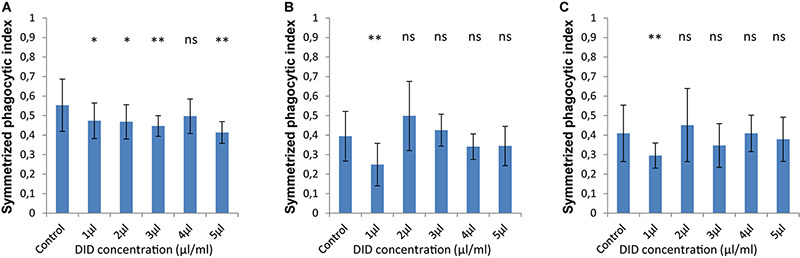
The symmetrized phagocytic index varies according to the DID concentration used for MH-S cell labeling during phagocytosis of unlabeled spores of *L. corymbifera* and *A. fumigatus* phagocytosed by DID-stained MH-S cells. **(A–C)** are as in Figure 2. Three independent biological replicates were carried out. Statistical difference was always measured in comparison to the control experiment and was described as: “ns” means there is no significant difference, *p < 0.05, **p < 0.01, ***p < 0.001. The data represent mean ± standard deviation.

In order to accurately quantify phagocytosis measurements, it is essential to reliably identify the cell membranes of the immune cells and of the spores, so that the spores that are inside these immune cells can be precisely detected. Motivated by our previous observations, we applied various concentrations of DID-staining (1–5 μl/ml, according to the guidelines provided by the manufacturer, see section “Materials and Methods”) to MH-S cells in order to quantify the effect of this widely used staining method on the phagocytosis process. We found that staining the membrane of MH-S cells with DID showed various influence on their capability to phagocytose either the FITC-stained (Figure 3) or the label-free (Figure 4) spores of *L. corymbifera* and *A. fumigatus* compared to unstained MH-S cells.

The interaction between labeled MH-S cells and FITC-stained spores showed a notable decrease in the phagocytosis measures as follows: For *A. fumigatus*, staining MH-S cells with various concentrations of DID (1–5 μl/ml) significantly decreased the value of phagocytosis of the FITC-stained spores by about 15–20% compared to label-free MH-S cells (Figure 3A). For the stained spores of the attenuated *L. corymbifera* strain JMRC: FSU: 10164, staining MH-S cells with 1 μl/ml DID concentration did not change the phagocytic capability of MH-S cells toward FITC-stained spores compared to unstained MH-S cells. However, increasing the staining of the MH-S cells to 2–5 μl/ml of DID significantly decreased the capability of MH-S cells to phagocytose FITC-stained spores (Figure 3B). Overall, depending on the DID concentration the phagocytosis was reduced by about 40% (Figure 3B) with variations in the phagocytosis measure. For the virulent strain of *L. corymbifera* JMRC: FSU: 09682, staining of MH-S cells with 1 and 2 μl/ml of DID didn’t change the phagocytic measure, however, higher concentrations (3–5 μl/ml of DID) induced a reduction of approximately 30% compared to the unstained samples (Figure 3C). In summary, staining the MH-S cell membrane with DID manipulates the capability of MH-S cells to phagocytose FITC-stained spores and the difference in this effect depends both on the concentration of the DID stain for MH-S cells as well as on the fungal strain used in the assay.

For unlabeled spores of *A. fumigatus*, the interaction between the MH-S cells labeled with various concentrations of DID (1–5 μl/ml) the symmetrized phagocytic index decreased during the confrontation assay (Figure 4A). Statistical analysis showed a significant difference in the phagocytic measures with all concentrations of DID except at 4 μl/ml when compared to unlabeled MH-S cells. On the contrary, the interaction between the DID-labeled MH-S cells and the label-free spores of the two strains of *L. corymbifera* showed no significant decrease in the symmetrized phagocytic index at all DID concentrations except at 1 μl/ml (Figures 4B,C). These results shed light on the differential influence of the labeling on the phagocytic measurements and that the underlying binding events at the molecular level must involve non-linear interactions yielding a non-intuitive dependence on the degree of DID staining.

### Staining Fungal Spores With FITC Enhances Phagocytosis by Unstained MH-S Cells

**FIGURE 5:**
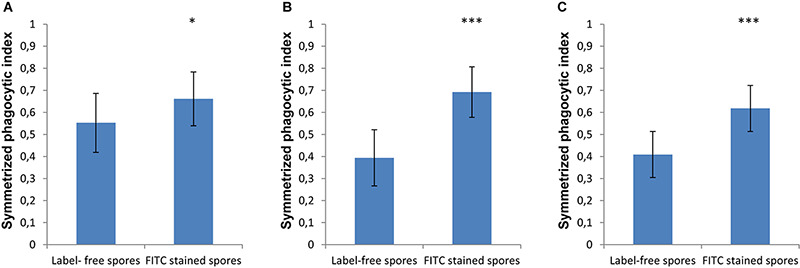
The effect of FITC-labeling on the phagocytosis process of *L. corymbifera* and *A. fumigatus* spores engulfed by unlabeled MH-S cells. **(A)** Symmetrized phagocytic index for *A. fumigatus* strain ATCC 46645. **(B)** Symmetrized phagocytic index for the attenuated *L. corymbifera* strain JMRC: FSU: 10164. **(C)** Symmetrized phagocytic index for a virulent *L. corymbifera* strain JMRC: FSU: 09682. Three independent biological replicates were carried out. Statistical difference was described as: “ns” means there is no significant difference, *p < 0.05, **p < 0.01, ***p < 0.001. The data represent mean ± standard deviation.

In order to monitor the spores that are phagocytosed by immune cells, the spores are typically labeled with FITC, which is a dye that is simple to apply, bright and photostable ensuring easy and reproducible microscopy experiments. However, in our current study we found that labeling spores with FITC increased the phagocytosis by MH-S cells of spores for both strains of *L. corymbifera* and for *A. fumigatus* compared to the label-free spores (Figure 5). Staining the spores of *A. fumigatus* with FITC increased their recognition by MH-S cells by 35% (Figure 5A). Moreover, for the spores of an attenuated strain of *L. corymbifera* JMRC: FSU: 10164, the uptake by MH-S cells was boosted by 75% when using FITC-labeled spores compared to label-free spores (Figure 5B). Interestingly, phagocytosis of the spores of the more virulent *L. corymbifera* strain JMRC: FSU: 09682 were enhanced for FITC-stained spores by only 50% (Figure 5C). The statistical analysis showed significant differences in the phagocytosis measures between the FITC-stained and label-free spores for all three fungal strains. Furthermore, the result showed that, even though this effect seems to have the same trend for all these strains of fungi with increases between 35 and 75% for FITC-labeled spores, the effect of FITC-staining can reverse the conclusions with regard to phagocytic vulnerability as compared to host–pathogen interactions in the unstained case. However, the FITC staining itself didn’t cause an appreciable change in the surface roughness of the spores, as shown by comparing the roughness values between FITC- and CFW-co-labeled spores with spores that were labeled only with CFW (Supplementary Figure S7).

### DID- and FITC-Staining Alter the Per-Macrophage Distribution of Phagocytosed and Adherent Spores

Imaging provides the full spatial information of confrontation assays and enables us to investigate whether the effect of membrane labeling is limited to the process of phagocytosis or also involves the adherence of the spores to the MH-S cell membranes. Our custom-written automated image analysis tool yielded the per-macrophage numbers of phagocytosed and adherent spores, which allowed us to calculate the probability density function (PDF) that characterizes the per-macrophage likelihood of having a certain number of spores either engulfed or adhered to the MH-S cells. Here we used all per-macrophage data of each fungal strain (i.e., we pooled all three replicates per fungal pathogen), thus resulting in a database from which we could calculate the probability of a macrophage having a certain number of spores either phagocytosed or adherent. The resulting PDF characterized each strain under every condition and was plotted as a bar graph.

**FIGURE 6:**
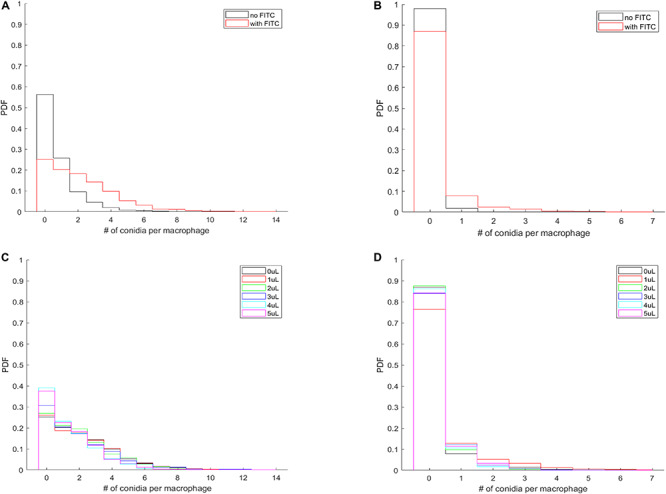
The per-macrophage distribution of phagocytosed and adherent spores is altered by FITC-labeling of *L. corymbifera* JMRC: FSU: 10164 confronted by unlabeled MH-S cells. **(A)** Probability density function (PDF) of the per-macrophage number of phagocytosed pathogens with and without FITC staining of the spores. **(B)** PDF for the distribution of per-macrophage adherent spores, otherwise the same conditions as in **(A)**. For **(A,B)**, black line: unlabeled spores; red line: FITC-labeled spores. **(C,D)** Effect of labeling the MH-S cells with various concentrations of DID on the PDF of phagocytosed and adherent FITC-labeled spores of *L. corymbifera* JMRC: FSU: 10164. **(C)** PDF for the per-macrophage number of phagocytosed spores. **(D)** PDF for the adherent spores. The DID concentrations of the MH-S cell labeling are plotted from 0 to 5 μl/ml in black, red, green, blue, cyan, and magenta, respectively.

The most natural behavior of the confrontation process is revealed when neither component is labeled (i.e., no FITC-staining of the spores, and no DID-labeling of the MH-S cells). The PDF of this assay is shown for *L. corymbifera* JMRC: FSU: 10164 by the black curves in Figures 6A,B. Accordingly, we found that staining the spores with FITC not only increases the number of phagocytosed spores on a per-population level (Figure 5), but also on a per-macrophage level for *L. corymbifera* JMRC: FSU: 10164 (Figure 6A), *L. corymbifera* JMRC: FSU: 09682 (Supplementary Figure S8A) and *A. fumigatus* (Supplementary Figure S8C). We found that the FITC-labeling also reduces the number of adherent spores of *L. corymbifera* JMRC: FSU: 10164 (Figure 6B), *L. corymbifera* JMRC: FSU: 09682 (Supplementary Figure S8B) and *A. fumigatus* (Supplementary Figure S8D) compared to label-free spores during confrontation with unlabeled MH-S cells.

The most common situation is when labeled spores are used with labeled MH-S cells. Interestingly, we found that labeling both components has a less obvious effect on the phagocytosis than on the adherence for the attenuated strain of *L. corymbifera* 10164 (Figures 6C,D, respectively). On the other hand, phagocytosis also decreased for the virulent strain of *L. corymbifera* (Supplementary Figure S9A) and for *A. fumigatus* (especially for 3 μl/ml DID concentration; see Supplementary Figure S9C), concomitantly with an increased adherence (Supplementary Figures S9B,D, respectively). Furthermore, staining the cell membrane of MH-S cells with various concentrations of DID also affect the PDF of the phagocytosed and adherent spores. We found that the PDF values decrease with increasing DID concentrations for the attenuated strain of *L. corymbifera* 10164 (Supplementary Figure S10A), with such decrease being compensated by an increase of the PDF value at the zero spores per MH-S cell column. The per-macrophage number of phagocytosed spores does not appreciably change with the various DID concentrations for the spores of the virulent strain of *L. corymbifera* 09682 (Supplementary Figure S10C) and for *A. fumigatus* (Supplementary Figure S11A). The PDF values also indicate that the adherence of unlabeled spores of the two strains of *L. corymbifera* and of *A. fumigatus* to DID-labeled MH-S cells increases compared to unlabeled MH-S cells (Supplementary Figures S10B,D, S11B, respectively).

### The Area Under the Curve (AUC) Values of the PDF Data Closely Resemble the Behavior of the Symmetrized Phagocytic Index

**FIGURE 7:**
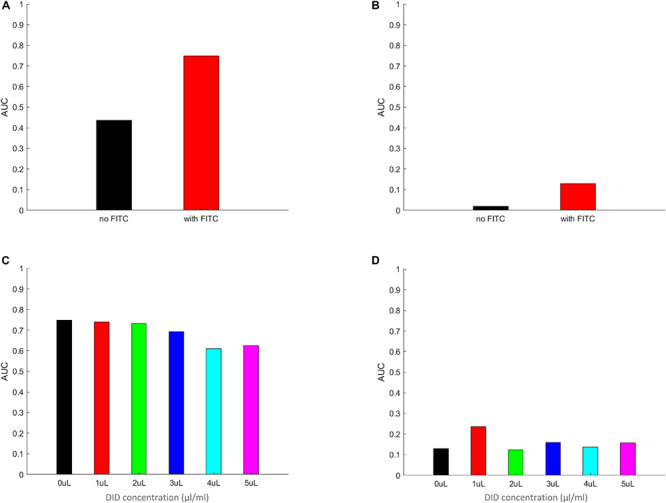
The area under the curve (AUC) of the PDF data from Figure 6. Panels **(A–D)** the AUC was calculated by multiplying the individual columns by the width of the column in the histograms in panels **(A–D)** in Figure 6, respectively. The zeroth columns (i.e., the MH-S macrophage cells without any adherent or phagocytosed spores) were left out of the integration. The resulting AUC values are plotted for FITC-free **(A)** and FITC-labeled **(B)** spores, as well for MH-S cells labeled with various concentrations of DID, **(C,D)**, applying the same color scheme as in Figure 6 for easier comparison between Figures 6, 7. The AUC calculated by using the equal weight of 1 for each column from Figure 6 results in a value that mimics the symmetrized phagocytic index of the corresponding fungal species and staining conditions.

By integrating the PDF functions, as calculated above, for the non-zero values of the per macrophages number of spores, we expect to receive a measure that closely mimics the symmetrized phagocytic index, but calculated based on per-macrophage data. In addition, we also gain information on the probability values of a spore being adherent, a new measure that the corresponds to the symmetrized phagocytic index but interpreted for adherent spores. The results of these calculations are summarized in Figure 7 and in Supplementary Figures S12–S15. Here, Figure 7 describes the AUC for the PDF data in Figure 6, whereas Supplementary Figures S12–S15 correspond to the PDF data in Supplementary Figures S8–S11, respectively. We noted a close resemblance between the AUC and the corresponding image-based symmetrized phagocytic indexes, both in absolute values and in trend. As an example, the comparison between Figures 5B, 7A and Figure 5C and Supplementary Figure S12A, as well as between Figure 5A and Supplementary Figure S12C (FITC-effect on the symmetrized phagocytic index for *L. corymbifera* 10164, 9682, and *A. fumigatus* ATCC, respectively) shows good agreement with respect to both the trend (*L. corymbifera* 10164 shows the largest FITC effect and *A. fumigatus* the smallest) and the absolute values. The same comparison carried out for the DID-effect on the symmetrized phagocytic index leads to a similar conclusion. In addition, we notice that FITC noticeably increases the adherence probability for both *L. corymbifera* strains (10164 and 9682 in Figure 7B and Supplementary Figure S12B, respectively). At the same time, DID-labeling increases the adherence probability of the *A. fumigatus* ATCC strain, Supplementary Figure S15B. Thus, the AUC results confirm our findings about the FITC- and DID effect on the phagocytosis probability, and it provides a new insight into the adherence probability of labeled and unlabeled spores when confronted with DID-labeled or unlabeled phagocytes.

In summary, the effect of membrane labels on the phagocytosis process is not limited to the engulfing step, but also involves affecting the adherence of the spores to the MH-S cell membranes. Our analysis tool has proven to be a promising tool to characterize such probabilities during immune system confrontation events.

## Discussion

Investigating the interaction between microorganisms and immune cells is a main subject of this study, in particular monitoring the phagocytosis process that is proceeded by the adhesion step between the host and pathogen cells surfaces (Hassan et al., 2019). Several studies were performed to find the most convenient method and technique that can be easily handled and saves lab consumables and experimental effort (Kraibooj et al., 2015; Cseresnyes et al., 2018; Hassan et al., 2019). FACS techniques and image-based analysis methods are the most common ways that are currently applied in the many laboratories. However, recent studies revealed that there are several advantages for the image-based analysis method over FACS analysis, because the former provides more precise phagocytic measurements (Zhou and Wong, 2006; Hassan et al., 2019). In our previous studies, we developed a user-friendly image analysis platform (ACAQ) that can automatically evaluate the phagocytic measures giving accurate results and decreasing the workload of manual analyses. Here we extended the ACAQ platform to determine the phagocytic measures without labeling either the host cells or the pathogens, except for a counter-staining that was necessary to distinguish between phagocytosed and non-phagocytosed spores (Mech et al., 2011, 2014; Kraibooj et al., 2014, 2015; Mattern et al., 2015; Hassan et al., 2019). In the current study, the main goal was to show whether the fluorescence labeling of the host cells and pathogens influences the value of the phagocytic measures and to develop a platform that is easily usable for the non-professional user to monitor the confrontation process between unstained host cells and label-free pathogens. This provides the optimal method for monitoring the phagocytosis processes as it occurs under realistic conditions without any interference from membrane labeling.

The order of the fixation process was proven to be important in determining the phagocytic measures, because applying formaldehyde fixation first (i.e., before counter-staining) permitted the entrance of the CFW-stain into the macrophages, where it unwantedly labeled the already phagocytosed spores with blue color. The entry of CFW could be caused by a decreased integrity of the cell membrane and increased permeability for the intra-and extracellular molecules (Cheng et al., 2019). Using CFW-staining at first provides a viable option to identify adherent pathogens, due to the reduced possibility of erroneously staining of engulfed spores with blue color. It is worth to note that even under the ideal conditions of CFW application, 20% of the spore population was labeled blue despite being inside macrophages. Such erroneous staining may be due to some CFW-positive cells having been phagocytosed even after stopping the phagocytosis process with cold saline, or by some MH-S cell membrane leakage even before formaldehyde was applied.

Using DID to label live cells is one of the most common methods in cell biology, because DID is a lipophilic dye that will bind to the lipid components of the cell membrane and subsequently labels the entire cell (Mohtasebi et al., 2014). Various studies investigated the effect of DID staining on the biological function of cells and they found staining the mesenchymal stem cells with DID does not have either apoptotic or cytotoxic effects, neither does it influence cell differentiation and cell morphology [23]. However, DID staining also has disadvantages, including the reduction of the expression level of extracellular matrix glycosaminoglycan that plays a role in various biological processes such as cell signaling and cell adhesion, as well as the non-linear relationship between DID concentration and DID fluorescence intensity (Sutton et al., 2009; Rutten et al., 2010; Mohtasebi et al., 2014). Moreover, DID was utilized to monitor the proliferation of cancer cells and it was revealed that while it did not have an effect on the proliferation of the cells the intensity of DID labeling had an inverse relationship with the rate of cell division (Mohtasebi et al., 2014). However, DID could also be transferred from dead cells to neighboring live cells, which lead to erroneous readouts from the experiment, thus raising the need for a marker that indicates the viability of the cells (Yumoto et al., 2014). Additionally, perturbation of the bending elasticity of lipid bilayer membranes was noticed while implementing the lipophilic staining, although this change was found to be not statistically significant (Bouvrais et al., 2010). Furthermore, the migration of DID in co-cultured cells depends mainly on the type of the cells (Lehmann et al., 2016), which consequently points at a differential effect of DID staining toward various immune cells, possibly caused by a difference in the components of the lipid structures. Even when using lower concentrations of lipophilic dyes such as DID for monitoring the behavior of stem and leukemic cells, an increase of micro-environmental contamination during cell death or phagocytosis process was observed (Lassailly et al., 2010). To the best of our knowledge, the current study is the first to clarify the staining effect of a lipophilic dye on the capability of macrophages as a main compartment of the innate immune system to phagocytose strains of the most common opportunistic fungi. A recent study confirmed that the phagocytosis capacity of macrophages depends mainly on lipids rafts (Lévêque et al., 2018). Because DID labels the lipid structures of the cell membrane specifically, DID may modulate the phagocytosis process and reduce the capability of macrophages to endocytose fungal spores through masking PRRs. Our finding is consistent with previous results that revealed a manipulation of biological processes caused by DID staining. Moreover, our study encourages us to extend the automated image analysis in the future to enable the complete label-free analysis of various assays, also involving deep-learning approaches from the modern field of the Artificial Intelligence.

FITC is a fluorescence probe that primarily targets proteins and binds to their amino groups, where the FITC-labeled proteins migrate to the positive charge at higher speed than the unstained proteins (Maeda et al., 1969). Moreover, FITC binds to proteins via non-covalent bonds that modify the proteins through changing the function of their amino groups and subsequently causes errors in the readout of biological experiments (Maeda et al., 1969). Other studies showed that FITC has various disadvantages, for example its stability is rather low, and therefore it is necessary to implement other stabilizing compounds such as dextrans, which further change the immune response toward the pathogens (Pustylnikov et al., 2014). This confirms that the modification of the N-terminal group of proteins by FITC, and the quenching of FITC-stained proteins by other residues chaperone the recognition of stained ligands by their receptors and minimize the intensity of emitted fluorescence (Jorbágy and Király, 1966; Marmé et al., 2003; Breen et al., 2016). All of these findings indicate that FITC staining manipulates the phagocytosis process and causes an error during the determination of the phagocytic measures. Staining the bacterial cells *Bordetella pertussis* with FITC affects their recognition by neutrophils, as labeling the bacterial cells with FITC increased the adherence of bacterial cells to neutrophils and reduced their phagocytosis compared to bacterial cells that were genetically labeled by introducing the green fluorescent protein (GFP) (Weingart et al., 1999). This phenomenon was reversed during opsonization as the rate of phagocytosis of *B. pertussis* by neutrophils increased. This finding confirmed the role of FITC labeling in changing the behavior of bacterial cells during interaction with immune cells (Weingart et al., 1999). Interestingly, the same study revealed that the staining of bacterial cells with FITC caused a fivefold reduction in the activity of adenylate cyclase toxin. This study proved that FITC staining hinders the activity of at least one of the virulence factors that is present on the surface of bacterial cells during confrontation with innate immune cells such as neutrophils. In the current study, our findings concur with the previous results, indicating that labeling the pathogen cells with FITC alters the results compared to label-free interactions. However, in contrast to the results in the literature, our findings indicate that the FITC staining increased the phagocytosis of fungal spores of *A. fumigatus* and two strains of *L. corymbifera* by macrophages. This dissimilarity may be due to various reasons, including: (1) as opposed to the previous study, we utilized fungal spores, not bacterial cells; there are obvious differences between the two microorganisms, because bacteria belong to prokaryotes and fungi are eukaryotes. One of these differences lies in the cell wall components that exhibit different attitudes toward the staining process with FITC; (2) macrophages were used as representatives of immune cells in the current study, whereas the previous study used neutrophils. The PRRs that are present on the surface of these two types of immune cells are different. We may add that each immune cell will likely show a different reaction toward any two different microorganisms (Duggan et al., 2015). In terms of the FITC effect on the spore surface roughness, we found no appreciable difference between the FITC-labeled and FITC-free spores (Supplementary Figure S7). We investigated both the volume-normalized and the surface area-normalized measures by dividing the number of the surface triangles by either the volume or the surface area of the spore, respectively. However, we must note that the spatial resolution of a confocal microscope limits the reliability of such technique in revealing small differences of surface roughness amongst the various species and strains of fungal spores. A more suitable technique for future studies may be provided by atomic force microscopy (Zolock et al., 2006).

In summary, in the current study we are able show that the staining of immune cells and pathogen influences the apparent value of the phagocytic measures. We also provide an extension of our previous ACAQ algorithm in order to identify and count the host and pathogen cells without pre-confrontation fluorescence staining. Here we still needed the post-confrontation labeling with CFW, with the future aim being to be able to remove this necessity as well. This toolkit will vastly improve the quality of monitoring phagocytosis processes, at the same time saving time, effort and consumables in the laboratory; it also prevents artificial readouts of the phagocytic measures that are caused by staining processes. Furthermore, our study provides an opportunity to reproduce the phagocytosis process more realistically as it takes place *in vivo*. These insights will be useful in various biological applications as it provides evidence about the effect of labeling in the recognition of pathogenic diseases by immune cells and offers computerized tools to identify and quantify biological living organisms without staining.

## Data Availability Statement

The datasets generated for this study are available on request to the corresponding author.

## Author Contributions

ZC designed the study, performed automated analysis of microscopy images, analyzed and interpreted the data, and wrote the manuscript. MH cultivated the spores and performed microscopy experiments, analyzed and interpreted the data, and wrote the manuscript. H-MD provided MH-S macrophages and advised on DID labeling. KV supervised MH, analyzed and interpreted the data, and provided experimental resources. MF conceived and designed the study, analyzed and interpreted the data, provided computational resources, and wrote the manuscript. All authors read and approved the final version of the manuscript.

## Conflict of Interest

The authors declare that the research was conducted in the absence of any commercial or financial relationships that could be construed as a potential conflict of interest.

## Footnotes

1 https://www.who.int/topics/infectious_diseases/en/

## Supplementary Material

FIGURE S1 Illustrative three-dimensional reconstructions of labeled and unlabeled MH-S cells, as well as labeled spores of *Lichtheimia corymbifera*. **(A–C)** Transmitted light images of unlabeled MH-S cells superimposed with green (FITC), blue (CFW) and combined (green and blue) spores of an attenuated *L. corymbifera* strain JMRC:FSU:10164, respectively. FITC-labeling was applied to the entire population of spores, whereas CFW-labeling should only stain non-phagocytosed spores. **(D–F)** Spores only. **(G–I)** As in **(A–C)** but showing the antibody-labeled MH-S cells in red. The scalebars indicate 10 μm.

FIGURE S2 Two-dimensional images of MH-S cells together with spores of the virulent *L. corymbifera* strain JMRC:FSU:09682 following a confrontation assay. **(A)** The MH-S cells and the fungal spores imaged in transmitted-light bright-field (TL-BF) microscopy mode of the confocal microscope. **(B)** The MH-S cells after staining with 5 μl/ml DID and imaged with the 561 nm laser of the confocal microscope. **(C)** The fungal spores stained with CFW and imaged with the 405 nm laser of the confocal microscope. **(D)** MH-S cells were segmented based on the TL-BF image (i.e., in a label-free way) using Hessian filtering by the augmented ACAQ-v3 software. The yellow outlines indicate the segmentation results. **(E)** The fungal spores were also segmented without fluorescence labeling using the updated ACAQ-v3 software. The yellow outlines show the segmentation results. The scalebars indicate 10 μm.

FIGURE S3 The morphometric measures provided by ACAQ-v4 allow the separation of a wide range of host and pathogen cell types. Cells were analyzed using ACAQ-v4 and their phagocytic and morphometric measures were determined. The relationships between the area and the aspect ratio, as well as between the area and the solidity are shown in **(A,B)**, respectively. The color and the shape of the symbols correspond to the cell types, as indicated by the legend on the top right area of the panels: alveolar macrophages (MH-S), bone marrow derived macrophages (BMDM), *Aspergillus fumigatus* (*A. fumigatus*), *Lichtheimia corymbifera* (*L. corymbifera*; data from three strains), *Mucor circinelloides* (*M. circinelloides*; data from six strains), *Saccharomyces cerevisiae* (*S. cerevisiae*; data from six strains). The symbols represent the mean value, whereas the horizontal and vertical bars indicate the standard deviation. Here solidity is defined as the ratio of the actual area of the object divided by the area of the convex hull.

FIGURE S4 Three-dimensional surface reconstructions of MH-S cells, FITC-labeled spores and CFW-counter stained spores of a virulent *L. corymbifera* strain JMRC: FSU: 09682. **(A,B)** Effect of counter-labeling the spores with CFW before MH-S cell fixation with formaldehyde **(A)** shows the green fluorescence channel that represents the FITC-stained spores, together with the red channel that corresponds to the MH-S cell membrane labeling that was used to outline the intracellular space of the macrophages, where the engulfed spores stained with FITC were located. The blue color labels the spores that were CFW-positive. **(B)** The majority of the CFW-positive (blue) cells are outside the macrophages. **(C,D)** Reconstructed results of the same staining protocol as in **(A,B)**, but for an experiment where the MH-S cells were first fixed with formaldehyde and then the spores were counter-labeled with CFW. **(C)** Combination of the green and red channels. **(D)** Most of the spores were also CFW-positive, not only FITC-labeled, thus revealing that the engulfed spores inside the MH-S cells also contain (erroneous) CFW-labeling.

FIGURE S5 **(A–D)** As in Supplementary Figure S1, but with the spores of an attenuated *L. corymbifera* strain JMRC:FSU:10164.

FIGURE S6 **(A,D)** As in Supplementary Figure S1, but with the spores of *A. fumigatus* strain ATCC 46645.

FIGURE S7 The surface roughness of FITC-labeled and FITC-free spores- Fungal spores of the two *Lichtheimia* species and of the *A. fumigatus* strain ATCC were imaged in three dimensions (*X*, *Y*, *Z*) with the same confocal microscope as above. The individual spores were reconstructed as 3D surfaces in Imaris 9.5.2. and morphometric measures were calculated by Imaris for each reconstructed spore. The number of the surface triangles were divided by either the volume **(A)** or the area **(B)** of the spore, in order to provide an estimate of the surface roughness of the spores. The experiments were carried out on spores with either only CFW-staining (columns labeled as “CFW:strain”), or co-labeled with CFW and FITC (columns labeled as “FITC:strain”). For each of the six data columns, images were acquired from three technical replicates per species, resulting in between 800 and 2,000 reconstructed spores per data column (total number of analyzed spores: 7300). The Cohen’s effect size of the difference between FITC-labeled and –unlabeled spores was as follows. For volume-based roughness values **(A)**: 0.28 (*L. corymbifera* 9682), 0.27 (*L. corymbifera* 10164), 0.45 (*A. fumigatus* ATCC). For area-based roughness values **(B)**: 0.05 (*L. corymbifera* 9682), 0.31 (*L. corymbifera* 10164), 0.63 (*A. fumigatus* ATCC). These effect sizes indicate low to medium biological significance for the FITC effect on the surface roughness. The data are represented as mean ± standard deviation.

FIGURE S8 **(A–D)** As in Figures 6A,B, but for FITC-labeled spores of a virulent *L. corymbifera* strain JMRC:FSU:09682 and *A. fumigatus* strain ATCC 46645, respectively.

FIGURE S9 **(A–D)** As in Figures 6C,D, but for FITC-labeled spores of a virulent *L. corymbifera* strain JMRC:FSU:09682 and *A. fumigatus* strain ATCC 46645, respectively.

FIGURE S10 The effect of labeling the MH-S cells with various concentrations of DID on the PDF of phagocytosed and adherent label-free spores of *L. corymbifera* strains. **(A,B)** PDF for the per-macrophage number of 10164 phagocytosed and adherent spores, respectively. **(C,D)** As for **(A,B)** but for the strain 9682. The DID concentrations of the MH-S cells labeling are plotted from 0 to 5 μl/ml in black, red, green, blue, cyan, and magenta, respectively.

FIGURE S11 The effect of labeling the MH-S cells with various concentrations of DID on the PDF of phagocytosed and adherent label-free spores of *Aspergillus fumigatus* strain ATCC 46645. **(A)** and **(B)**: PDF for the per-macrophage number of phagocytosed and adherent spores spores of *Aspergillus fumigatus* strain ATCC 46645, respectively.

FIGURE S12 The area under the curve (AUC) of the PDF data from Supplementary Figure S8. **(A–D)** The AUC was calculated by multiplying the height of the individual columns by the width of the column in the histograms in panels **(AD)** in Supplementary Figure S8, respectively. The zeroth columns (i.e., the MH-S macrophage cells without any adherent or phagocytosed spores) were left out of the integration. The FITC-free and FITC-labeled spores in **(A–D)** are labeled in black and red, accordingly. The AUC was calculated by using the equal weight of 1 for each column from Supplementary Figure S8, thus the AUC values mimic the symmetrized phagocytic index of the corresponding fungal species and staining conditions.

FIGURE S13 The area under the curve (AUC) of the PDF data from Supplementary Figure S9. **(A)** through **(D)**: the AUC was calculated by multiplying the height of the individual columns by the width of the column in the histograms in panels **(A)** through **(D)** in Supplementary Figure S9, respectively. The zeroth columns (i.e., the MH-S macrophage cells without any adherent or phagocytosed spores) were left out of the integration. The FITC-free and FITC-labeled spores in **(A)** through **(D)** are labeled in black and red, accordingly. The AUC was calculated by using the equal weight of 1 for each column from Supplementary Figure S9, thus the AUC values mimic the symmetrized phagocytic index of the corresponding fungal species and staining conditons. The color scheme of the columns depicting the various DID concentrations between 0 and 5 μl/ml is the same as used in the legends of Supplementary Figure S9.

FIGURE S14 The area under the curve (AUC) of the PDF data from Supplementary Figure S10. **(A)** through **(D)**: the AUC was calculated by multiplying the height of the individual columns by the width of the column in the histograms in panels **(A)** through **(D)** in Supplementary Figure S10, respectively. The zeroth columns (i.e., the MH-S macrophage cells without any adherent or phagocytosed spores) were left out of the integration. The FITC-free and FITC-labeled spores in **(A)** through **(D)** are labeled in black and red, accordingly. The AUC was calculated by using the equal weight of 1 for each column from Supplementary Figure S10, thus the AUC values mimic the symmetrized phagocytic index of the corresponding fungal species and staining conditons. The color scheme of the columns depicting the various DID concentrations between 0 and 5 μl/ml is the same as used in the legends of Supplementary Figure S10.

FIGURE S15 The area under the curve (AUC) of the PDF data from Supplementary Figure S11. **(A)** through **(D)**: the AUC was calculated by multiplying the height of the individual columns by the width of the column in the histograms in panels **(A)** through **(D)** in Supplementary Figure S11, respectively. The zeroth columns (i.e., the MH-S macrophage cells without any adherent or phagocytosed spores) were left out of the integration. The FITC-free and FITC-labeled spores in **(A)** through **(D)** are labeled in black and red, accordingly. The AUC was calculated by using the equal weight of 1 for each column from Supplementary Figure S11, thus the AUC values mimic the symmetrized phagocytic index of the corresponding fungal species and staining conditons. The color scheme of the columns depicting the various DID concentrations between 0 and 5 μl/ml is the same as used in the legends of Supplementary Figure S11.

VIDEO S1 Three-dimensional overview of the segmentation of FITC- and CFW-labeled spores inside MH-S cells (macrophage membrane in red). The animation shows that a large number of phagocytosed spores of a virulent *L. corymbifera* strain JMRC: FSU: 09682 inside MH-S cells were also stained with CFW (blue) due to applying the formaldehyde fixation step first followed later by CFW-staining.

VIDEO S2 As in Supplementary Video S1, but for a labeling order where CFW-staining was applied first, followed by the formaldehyde fixation.

VIDEO S3 As in Supplementary Video S1, but for an attenuated *L. corymbifera* strain JMRC: FSU: 10164.

VIDEO S4 As in Supplementary Video S2, but for an attenuated *L. corymbifera* strain JMRC: FSU: 10164.

VIDEO S5 As in Supplementary Video S1, but for *A. fumigatus* strain ATCC 46645.

VIDEO S6 As in Supplementary Video S2, but for *A. fumigatus* strain ATCC 46645.
