# Supplementary figures and images for: Quantitative Impact of Cell Membrane Fluorescence Labeling on Phagocytosis Measurements in Confrontation Assays
Source: Front Microbiol. 2020 Jun 5;11:1193. doi: 10.3389/fmicb.2020.01193 (PMC7289966; doi:10.3389/fmicb.2020.01193)

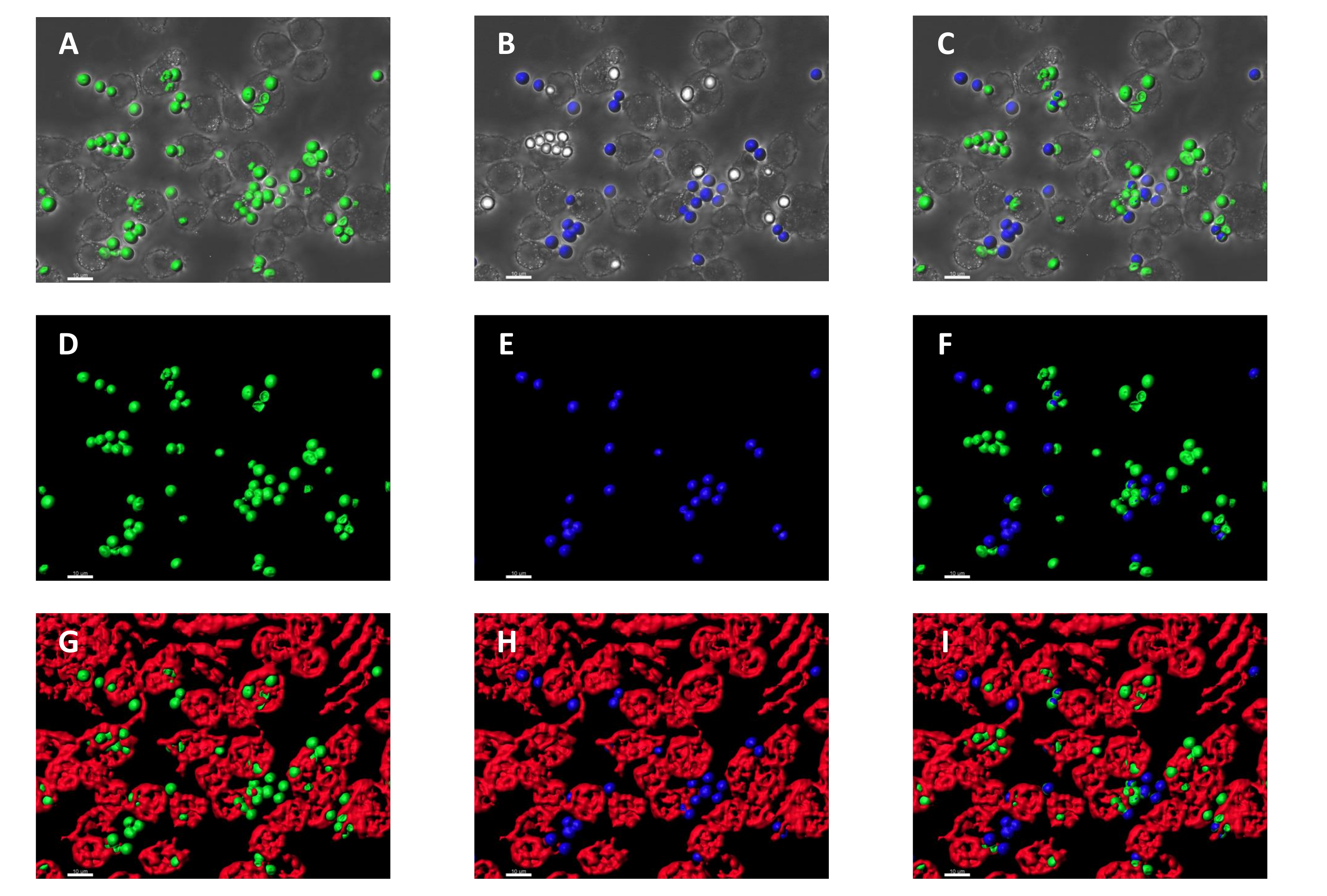

Supplement: FIGURE S1 — Illustrative three-dimensional reconstructions of labeled and unlabeled MH-S cells, as well as labeled spores of Lichtheimia corymbifera. (A–C) Transmitted light images of unlabeled MH-S cells superimposed with green (FITC), blue (CFW) and combined (green and blue) spores of an attenuated L. corymbifera strain JMRC:FSU:10164, respectively. FITC-labeling was applied to the entire population of spores, whereas CFW-labeling should only stain non-phagocytosed spores. (D–F) Spores only. (G–I) As in (A–C) but showing the antibody-labeled MH-S cells in red. The scalebars indicate 10 μm. [file Image_1.TIF]

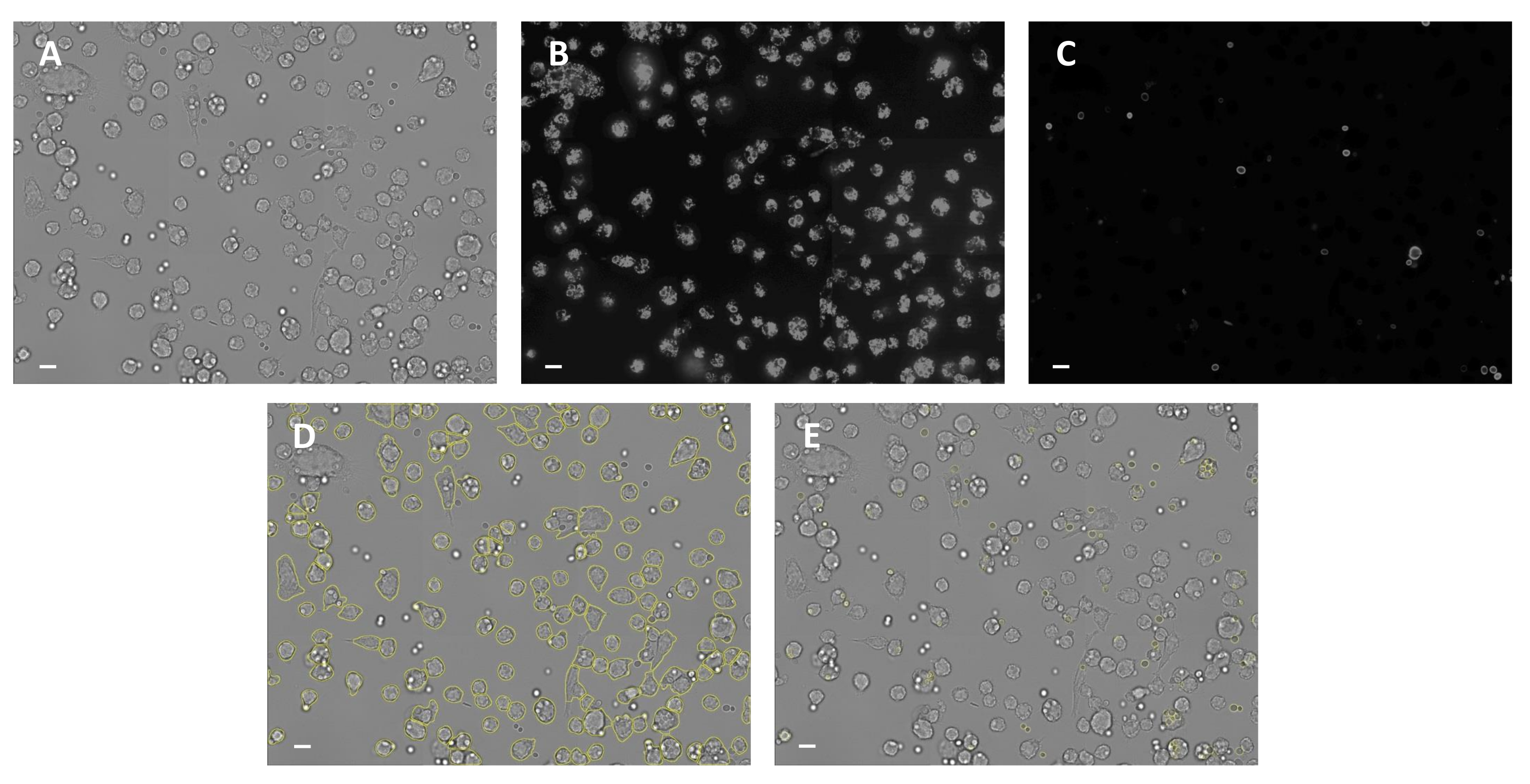

Supplement: FIGURE S2 — Two-dimensional images of MH-S cells together with spores of the virulent L. corymbifera strain JMRC:FSU:09682 following a confrontation assay. (A) The MH-S cells and the fungal spores imaged in transmitted-light bright-field (TL-BF) microscopy mode of the confocal microscope. (B) The MH-S cells after staining with 5 μl/ml DID and imaged with the 561 nm laser of the confocal microscope. (C) The fungal spores stained with CFW and imaged with the 405 nm laser of the confocal microscope. (D) MH-S cells were segmented based on the TL-BF image (i.e., in a label-free way) using Hessian filtering by the augmented ACAQ-v3 software. The yellow outlines indicate the segmentation results. (E) The fungal spores were also segmented without fluorescence labeling using the updated ACAQ-v3 software. The yellow outlines show the segmentation results. The scalebars indicate 10 μm. [file Image_2.TIF]

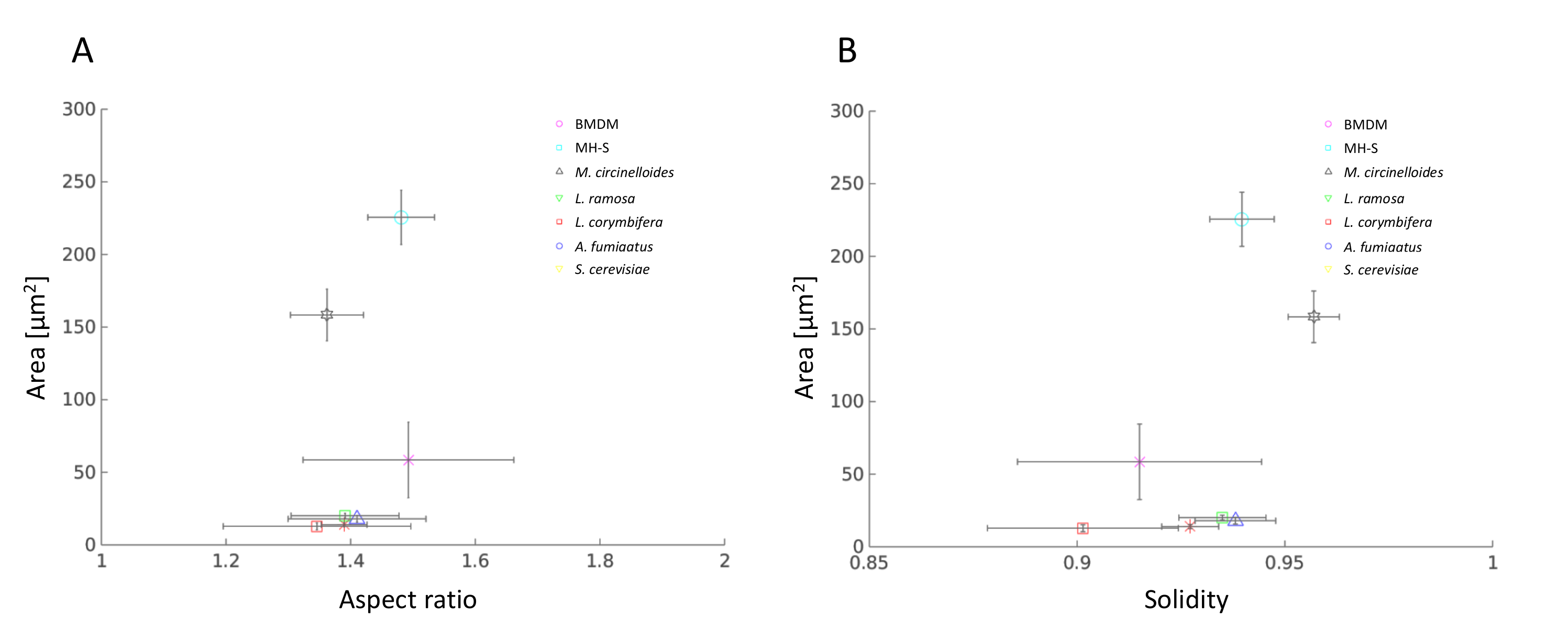

Supplement: FIGURE S3 — The morphometric measures provided by ACAQ-v4 allow the separation of a wide range of host and pathogen cell types. Cells were analyzed using ACAQ-v4 and their phagocytic and morphometric measures were determined. The relationships between the area and the aspect ratio, as well as between the area and the solidity are shown in (A,B), respectively. The color and the shape of the symbols correspond to the cell types, as indicated by the legend on the top right area of the panels: alveolar macrophages (MH-S), bone marrow derived macrophages (BMDM), Aspergillus fumigatus (A. fumigatus), Lichtheimia corymbifera (L. corymbifera; data from three strains), Mucor circinelloides (M. circinelloides; data from six strains), Saccharomyces cerevisiae (S. cerevisiae; data from six strains). The symbols represent the mean value, whereas the horizontal and vertical bars indicate the standard deviation. Here solidity is defined as the ratio of the actual area of the object divided by the area of the convex hull. [file Image_3.TIF]

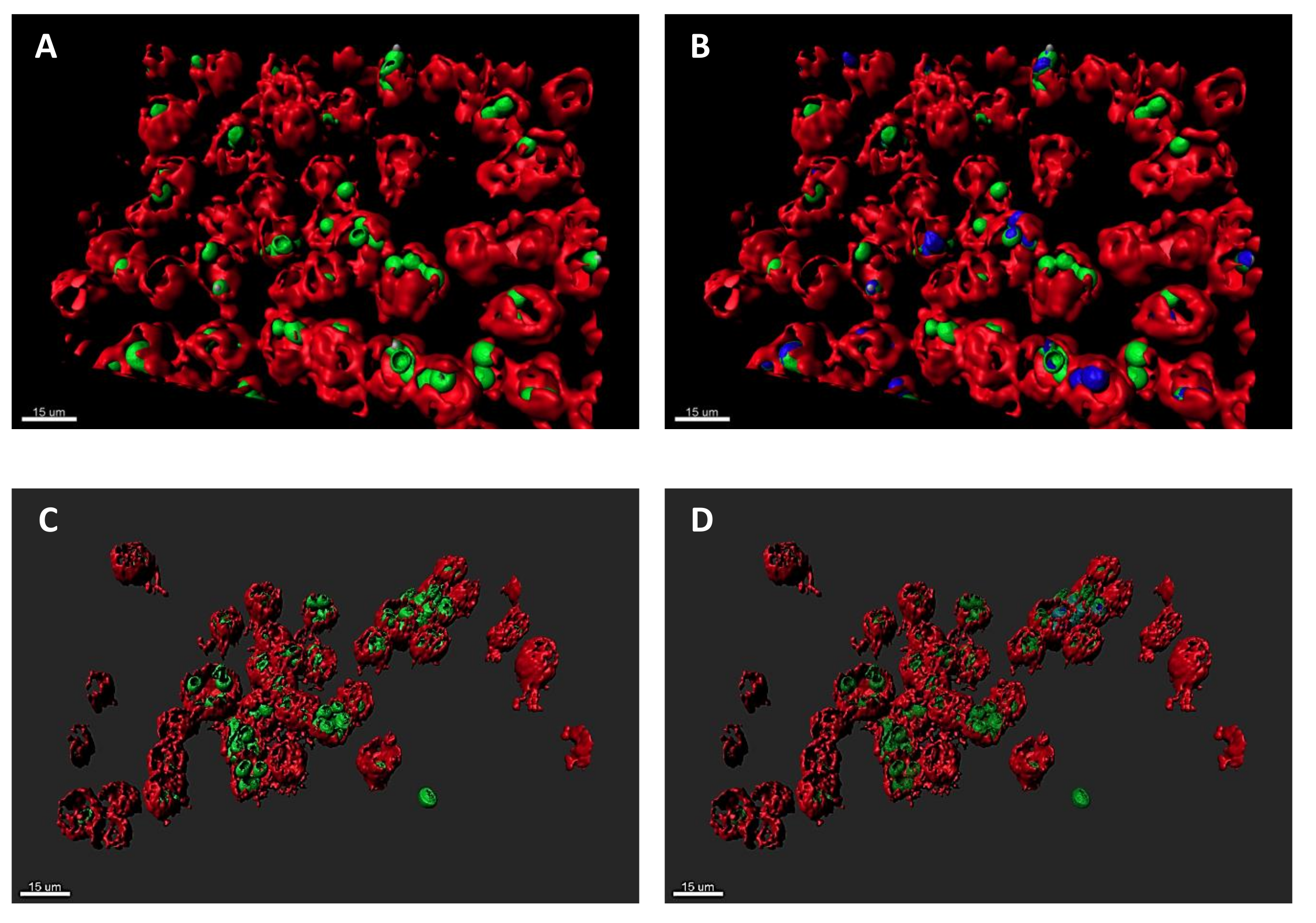

Supplement: FIGURE S4 — Three-dimensional surface reconstructions of MH-S cells, FITC-labeled spores and CFW-counter stained spores of a virulent L. corymbifera strain JMRC: FSU: 09682. (A,B) Effect of counter-labeling the spores with CFW before MH-S cell fixation with formaldehyde (A) shows the green fluorescence channel that represents the FITC-stained spores, together with the red channel that corresponds to the MH-S cell membrane labeling that was used to outline the intracellular space of the macrophages, where the engulfed spores stained with FITC were located. The blue color labels the spores that were CFW-positive. (B) The majority of the CFW-positive (blue) cells are outside the macrophages. (C,D) Reconstructed results of the same staining protocol as in (A,B), but for an experiment where the MH-S cells were first fixed with formaldehyde and then the spores were counter-labeled with CFW. (C) Combination of the green and red channels. (D) Most of the spores were also CFW-positive, not only FITC-labeled, thus revealing that the engulfed spores inside the MH-S cells also contain (erroneous) CFW-labeling. [file Image_4.TIF]

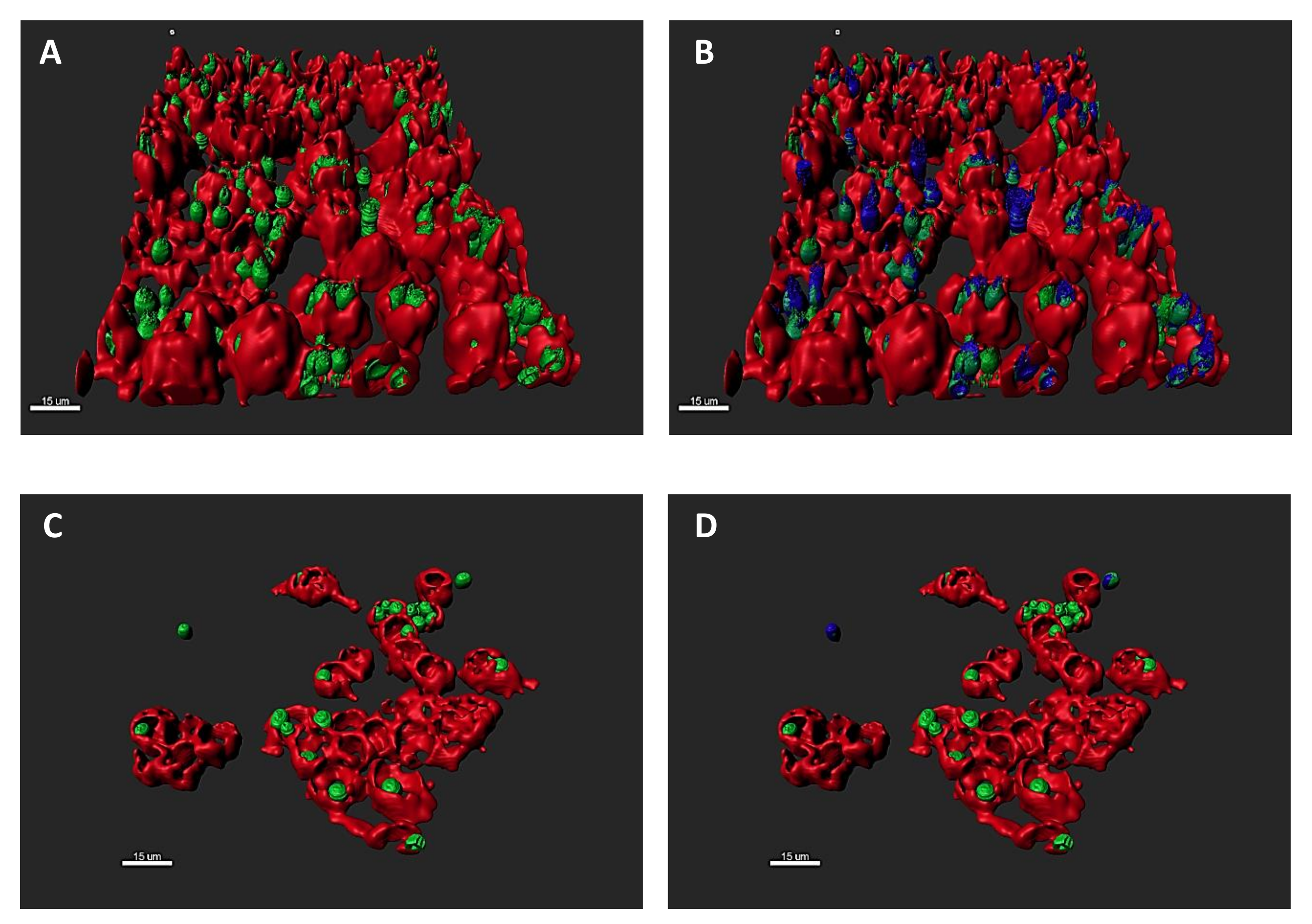

Supplement: FIGURE S5 — (A–D) As in Supplementary Figure S1, but with the spores of an attenuated L. corymbifera strain JMRC:FSU:10164. [file Image_5.TIF]

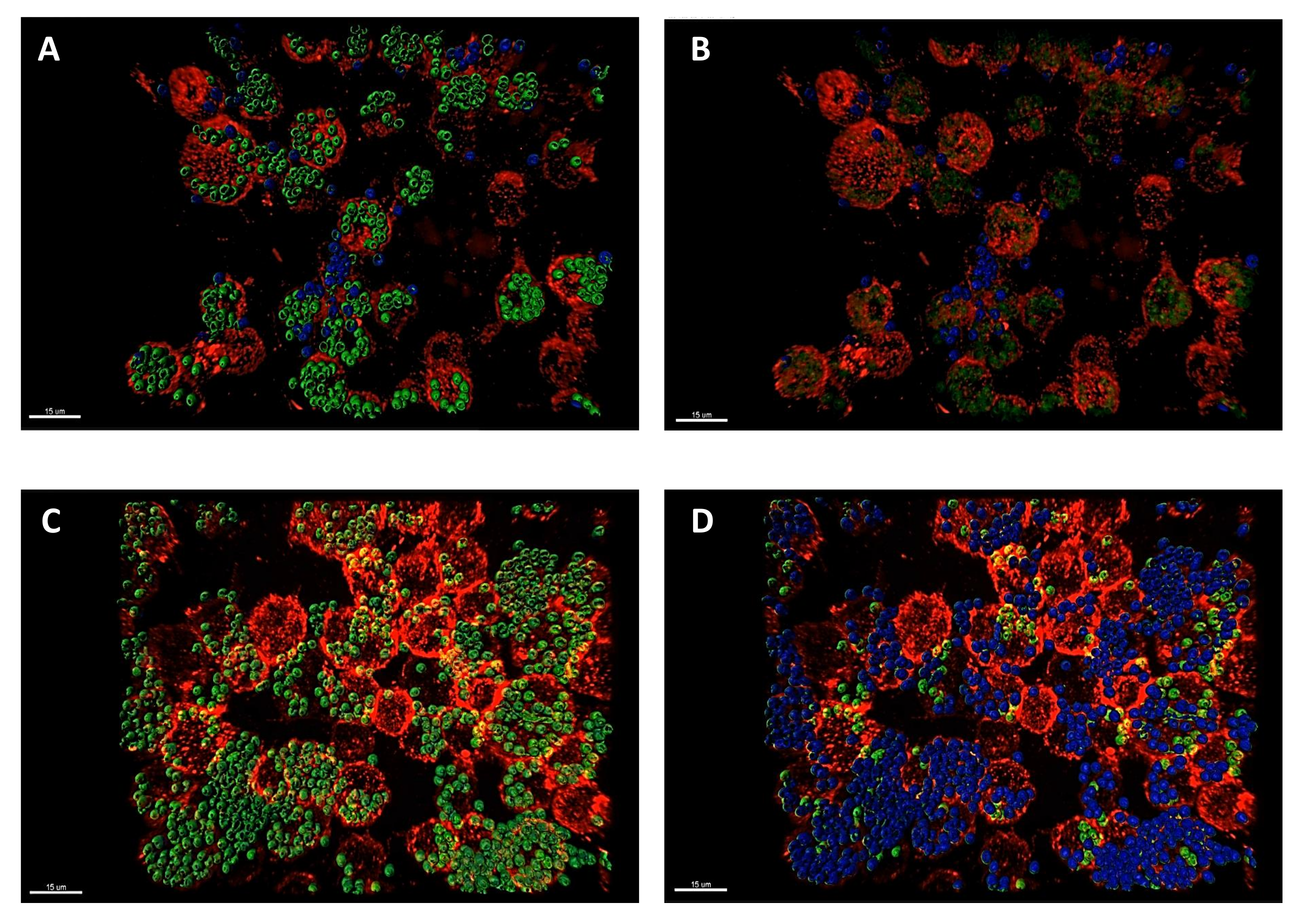

Supplement: FIGURE S6 — (A,D) As in Supplementary Figure S1, but with the spores of A. fumigatus strain ATCC 46645. [file Image_6.TIF]

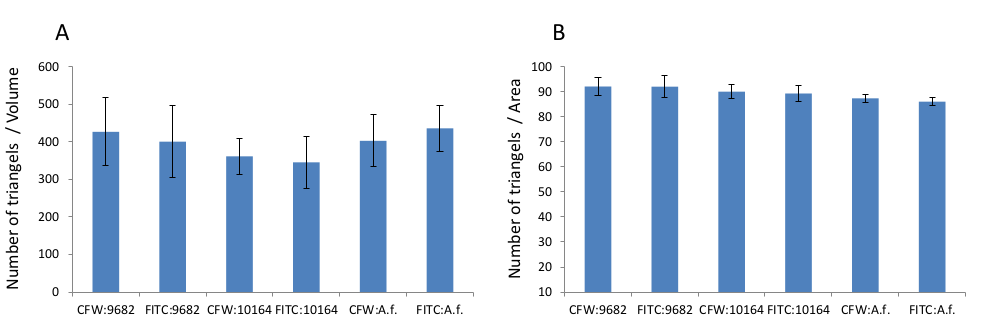

Supplement: FIGURE S7 — The surface roughness of FITC-labeled and FITC-free spores- Fungal spores of the two Lichtheimia species and of the A. fumigatus strain ATCC were imaged in three dimensions (X, Y, Z) with the same confocal microscope as above. The individual spores were reconstructed as 3D surfaces in Imaris 9.5.2. and morphometric measures were calculated by Imaris for each reconstructed spore. The number of the surface triangles were divided by either the volume (A) or the area (B) of the spore, in order to provide an estimate of the surface roughness of the spores. The experiments were carried out on spores with either only CFW-staining (columns labeled as “CFW:strain”), or co-labeled with CFW and FITC (columns labeled as “FITC:strain”). For each of the six data columns, images were acquired from three technical replicates per species, resulting in between 800 and 2,000 reconstructed spores per data column (total number of analyzed spores: 7300). The Cohen’s effect size of the difference between FITC-labeled and –unlabeled spores was as follows. For volume-based roughness values (A): 0.28 (L. corymbifera 9682), 0.27 (L. corymbifera 10164), 0.45 (A. fumigatus ATCC). For area-based roughness values (B): 0.05 (L. corymbifera 9682), 0.31 (L. corymbifera 10164), 0.63 (A. fumigatus ATCC). These effect sizes indicate low to medium biological significance for the FITC effect on the surface roughness. The data are represented as mean ± standard deviation. [file Image_7.TIF]

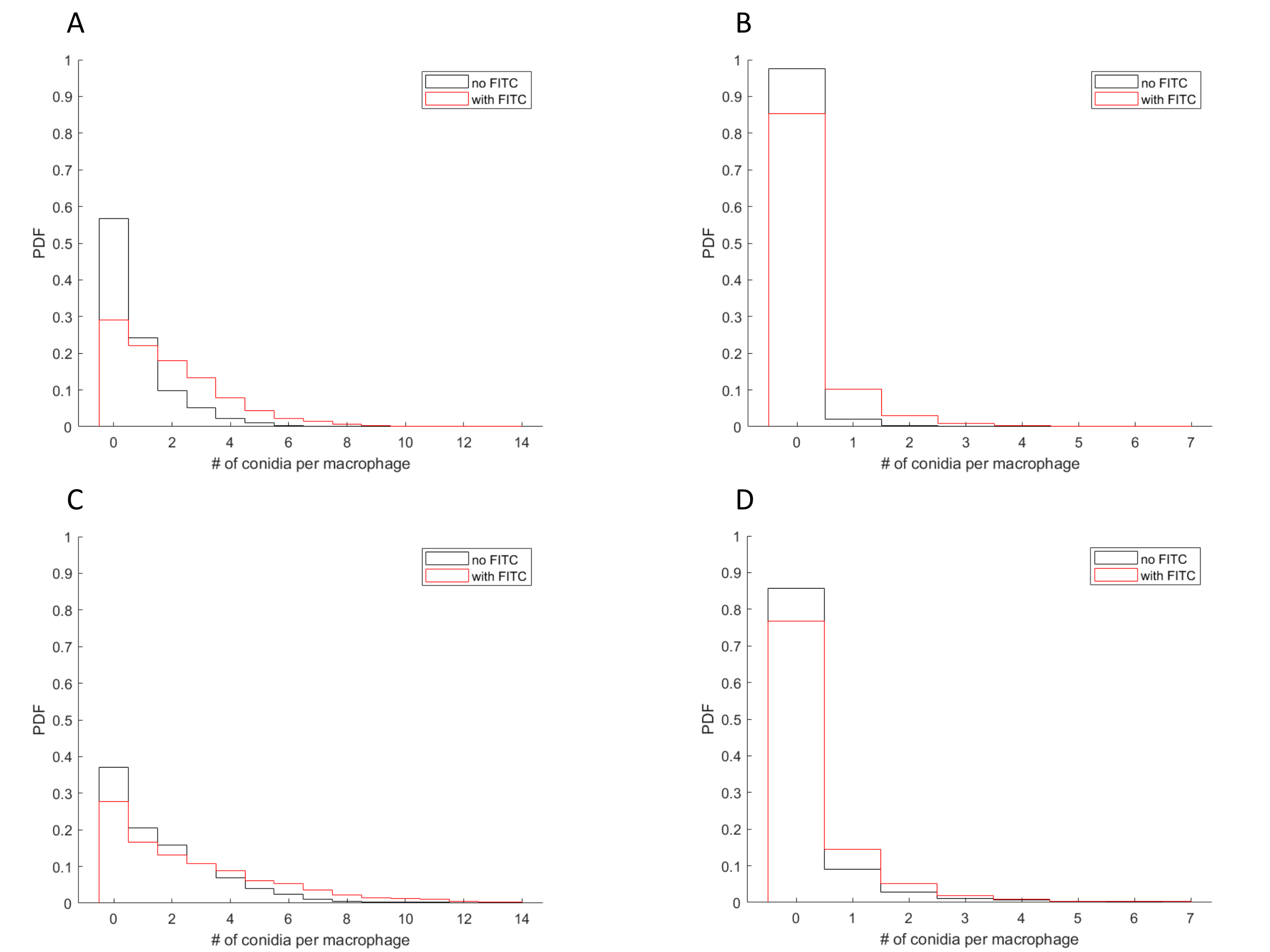

Supplement: FIGURE S8 — (A–D) As in Figures 6A,B, but for FITC-labeled spores of a virulent L. corymbifera strain JMRC:FSU:09682 and A. fumigatus strain ATCC 46645, respectively. [file Image_8.TIF]

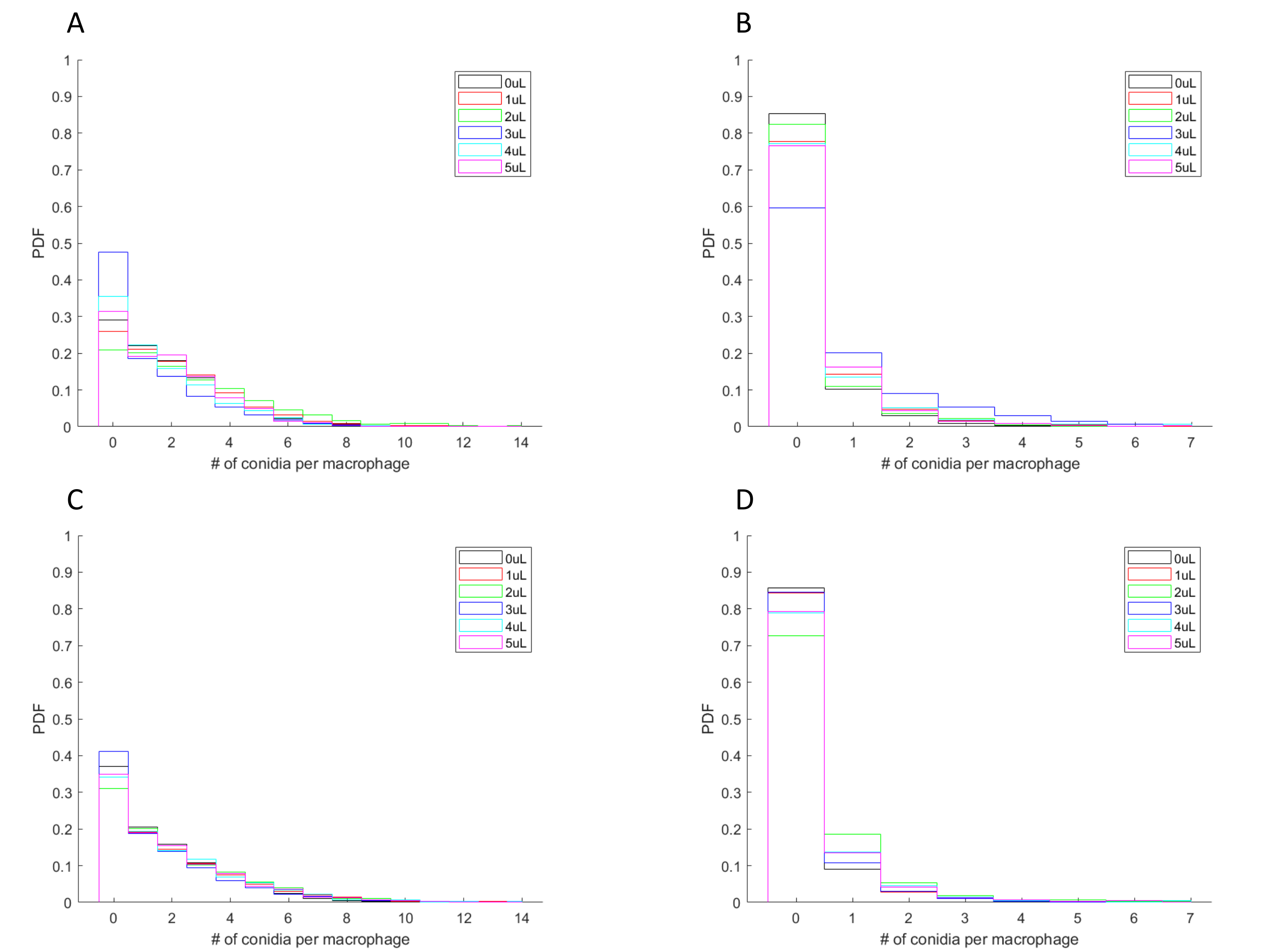

Supplement: FIGURE S9 — (A–D) As in Figures 6C,D, but for FITC-labeled spores of a virulent L. corymbifera strain JMRC:FSU:09682 and A. fumigatus strain ATCC 46645, respectively. [file Image_9.TIF]

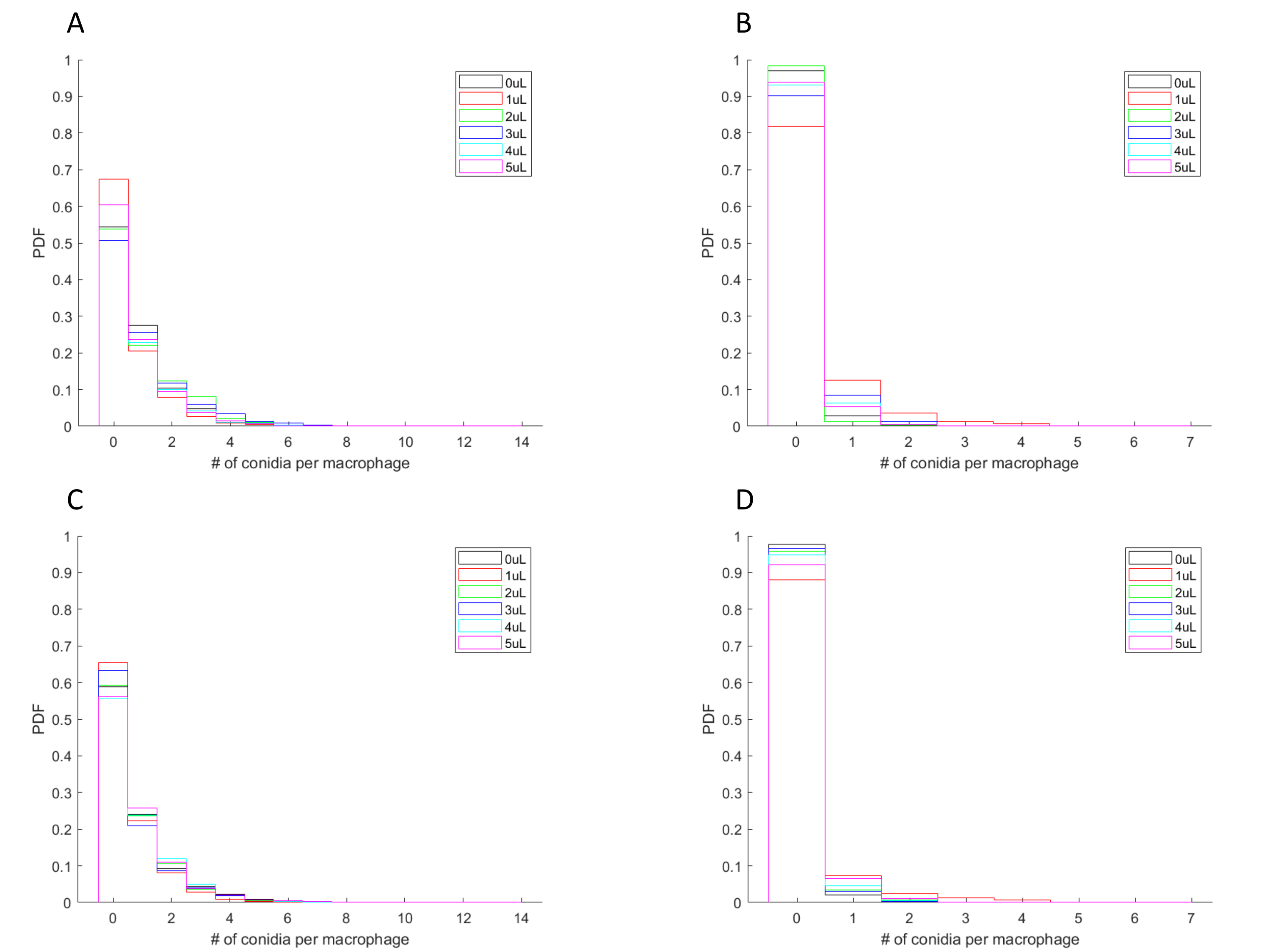

Supplement: FIGURE S10 — The effect of labeling the MH-S cells with various concentrations of DID on the PDF of phagocytosed and adherent label-free spores of L. corymbifera strains. (A,B) PDF for the per-macrophage number of 10164 phagocytosed and adherent spores, respectively. (C,D) As for (A,B) but for the strain 9682. The DID concentrations of the MH-S cells labeling are plotted from 0 to 5 μl/ml in black, red, green, blue, cyan, and magenta, respectively. [file Image_10.TIF]

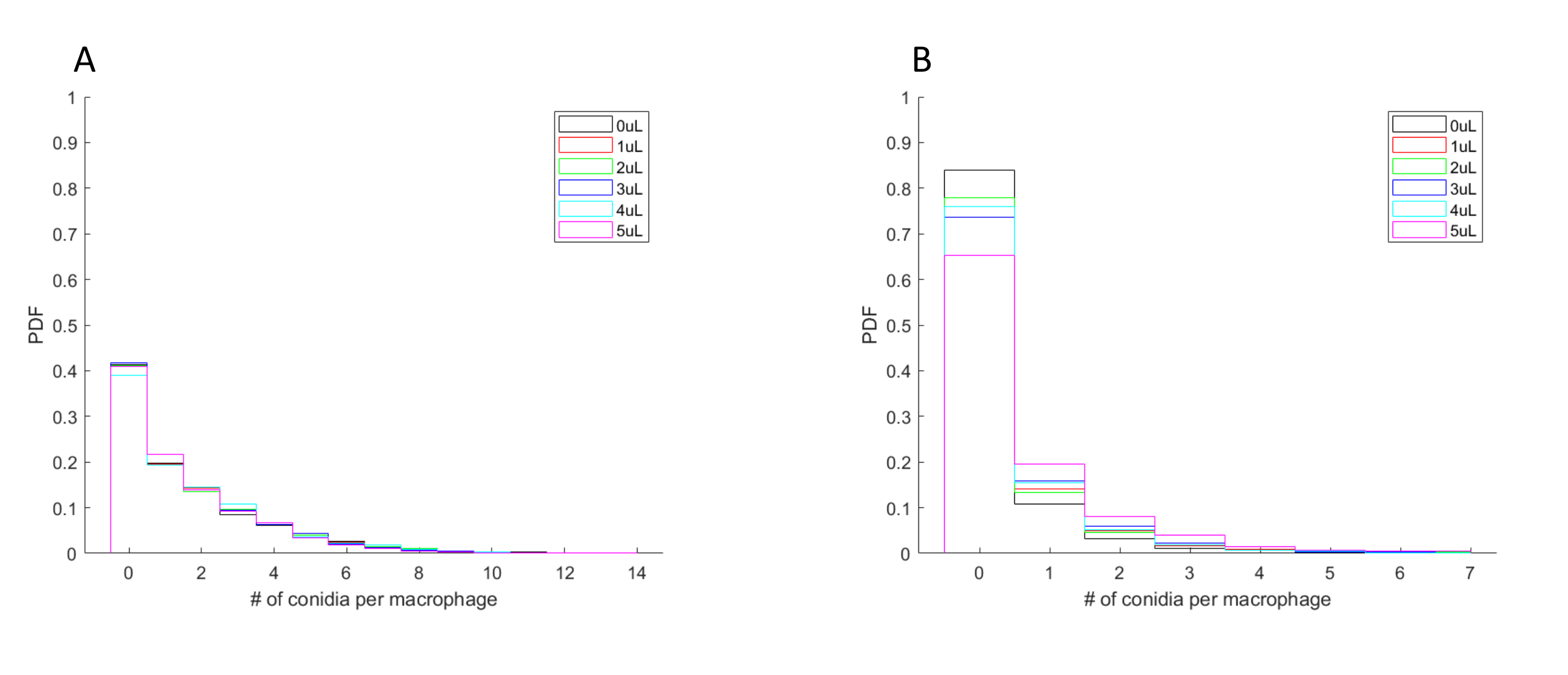

Supplement: FIGURE S11 — The effect of labeling the MH-S cells with various concentrations of DID on the PDF of phagocytosed and adherent label-free spores of Aspergillus fumigatus strain ATCC 46645. (A) and (B): PDF for the per-macrophage number of phagocytosed and adherent spores spores of Aspergillus fumigatus strain ATCC 46645, respectively. [file Image_11.TIF]

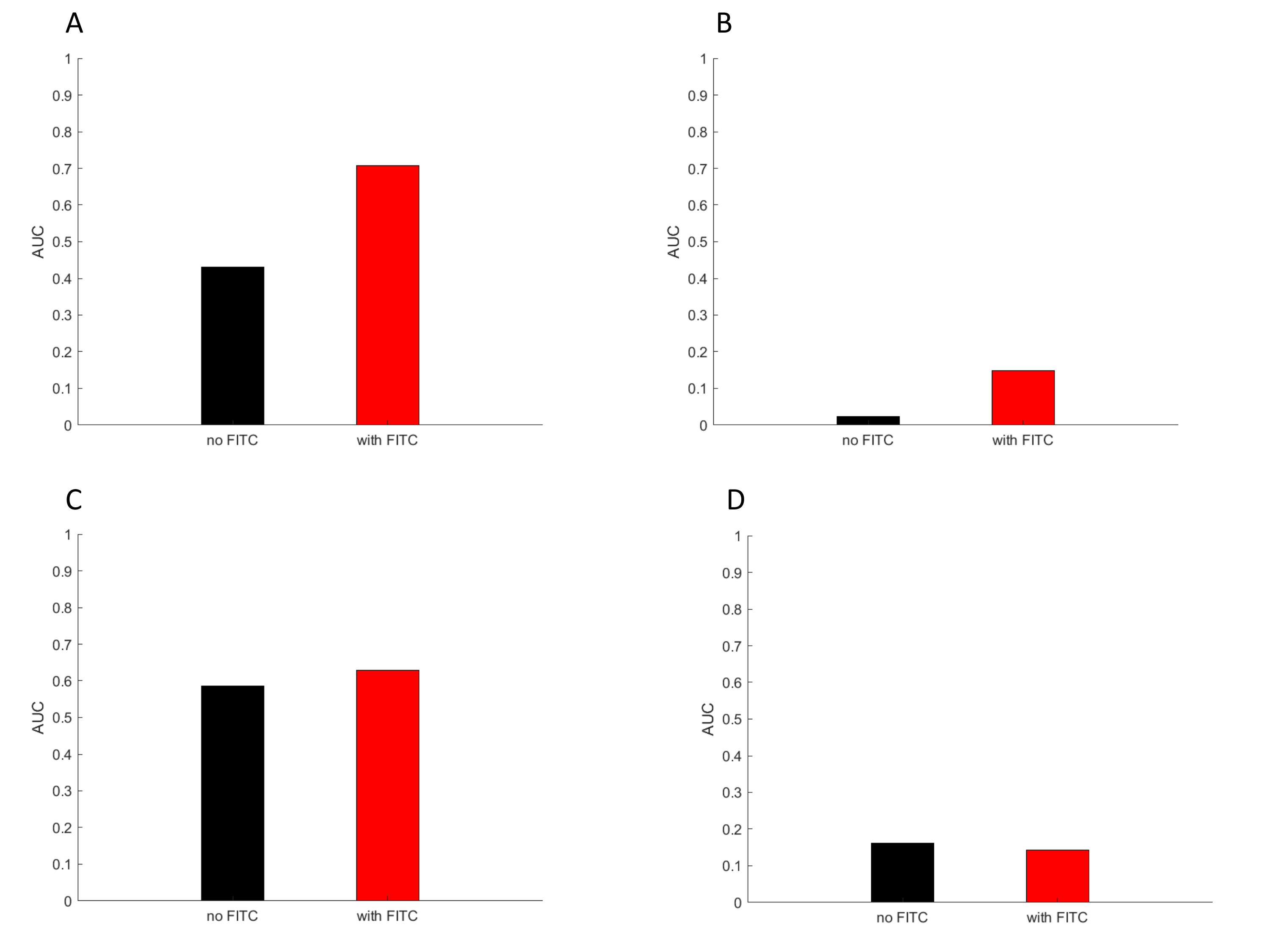

Supplement: FIGURE S12 — The area under the curve (AUC) of the PDF data from Supplementary Figure S8. (A–D) The AUC was calculated by multiplying the height of the individual columns by the width of the column in the histograms in panels (AD) in Supplementary Figure S8, respectively. The zeroth columns (i.e., the MH-S macrophage cells without any adherent or phagocytosed spores) were left out of the integration. The FITC-free and FITC-labeled spores in (A–D) are labeled in black and red, accordingly. The AUC was calculated by using the equal weight of 1 for each column from Supplementary Figure S8, thus the AUC values mimic the symmetrized phagocytic index of the corresponding fungal species and staining conditions. [file Image_12.TIF]

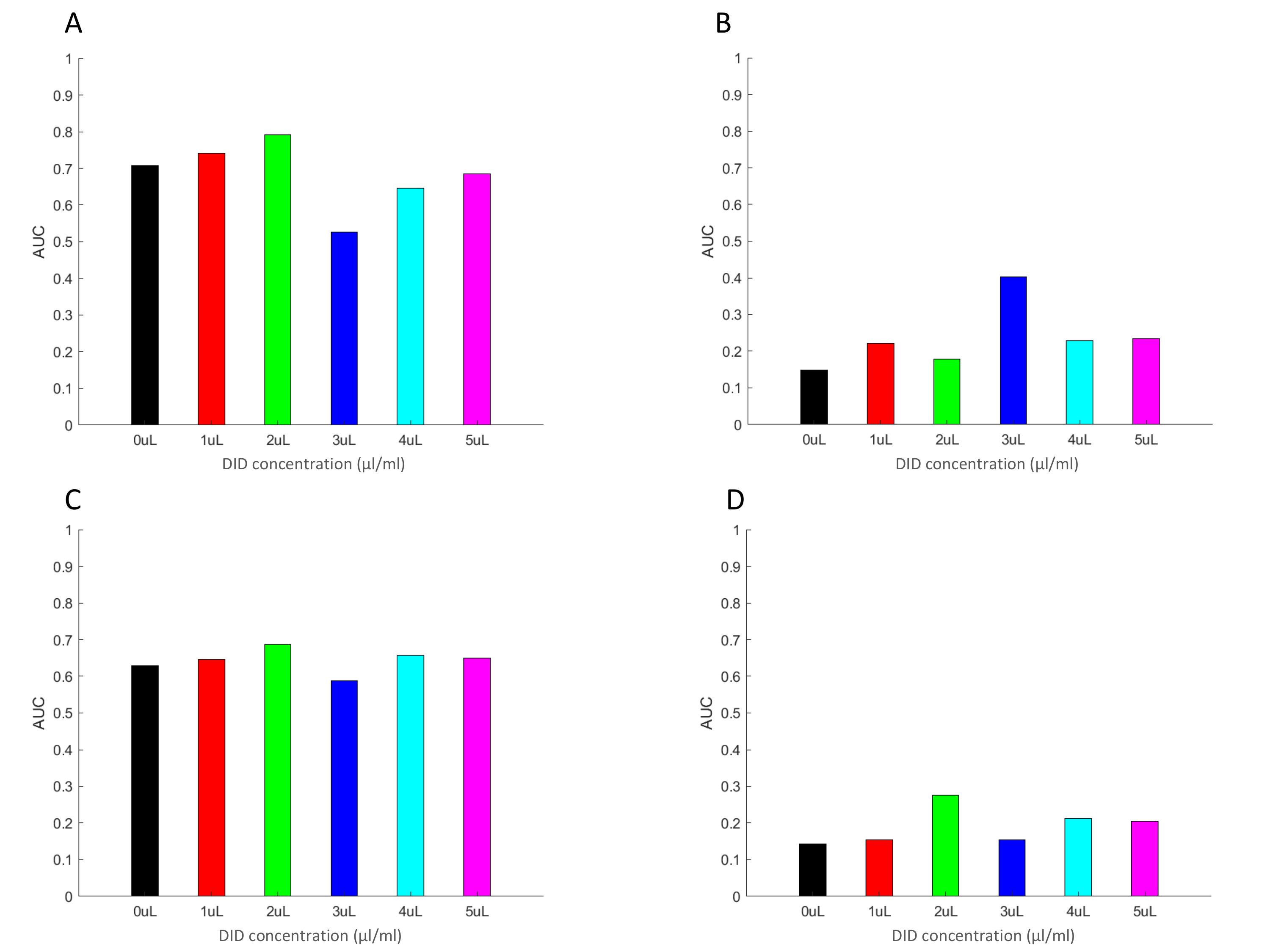

Supplement: FIGURE S13 — The area under the curve (AUC) of the PDF data from Supplementary Figure S9. (A) through (D): the AUC was calculated by multiplying the height of the individual columns by the width of the column in the histograms in panels (A) through (D) in Supplementary Figure S9, respectively. The zeroth columns (i.e., the MH-S macrophage cells without any adherent or phagocytosed spores) were left out of the integration. The FITC-free and FITC-labeled spores in (A) through (D) are labeled in black and red, accordingly. The AUC was calculated by using the equal weight of 1 for each column from Supplementary Figure S9, thus the AUC values mimic the symmetrized phagocytic index of the corresponding fungal species and staining conditons. The color scheme of the columns depicting the various DID concentrations between 0 and 5 μl/ml is the same as used in the legends of Supplementary Figure S9. [file Image_13.TIF]

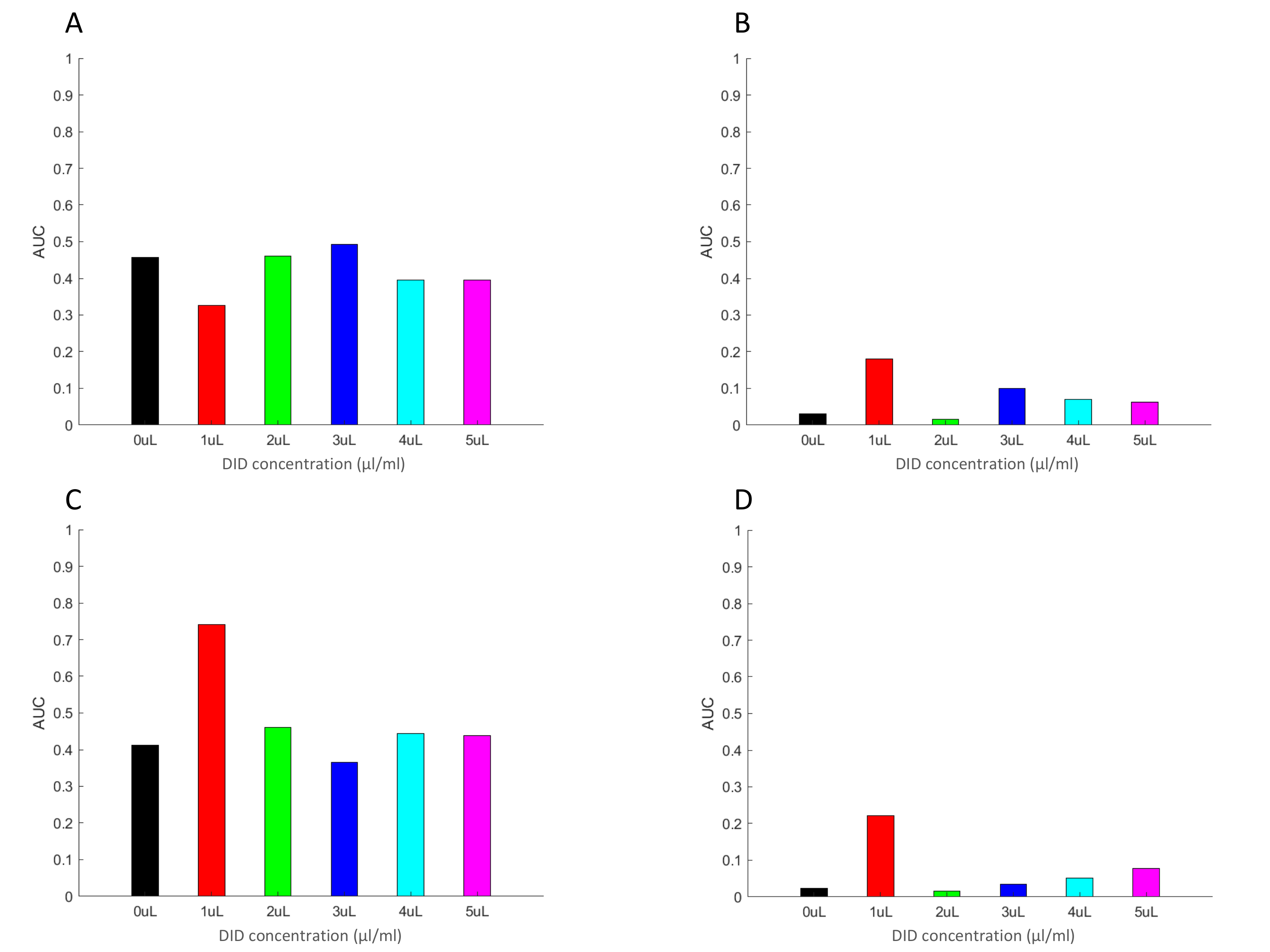

Supplement: FIGURE S14 — The area under the curve (AUC) of the PDF data from Supplementary Figure S10. (A) through (D): the AUC was calculated by multiplying the height of the individual columns by the width of the column in the histograms in panels (A) through (D) in Supplementary Figure S10, respectively. The zeroth columns (i.e., the MH-S macrophage cells without any adherent or phagocytosed spores) were left out of the integration. The FITC-free and FITC-labeled spores in (A) through (D) are labeled in black and red, accordingly. The AUC was calculated by using the equal weight of 1 for each column from Supplementary Figure S10, thus the AUC values mimic the symmetrized phagocytic index of the corresponding fungal species and staining conditons. The color scheme of the columns depicting the various DID concentrations between 0 and 5 μl/ml is the same as used in the legends of Supplementary Figure S10. [file Image_14.TIF]

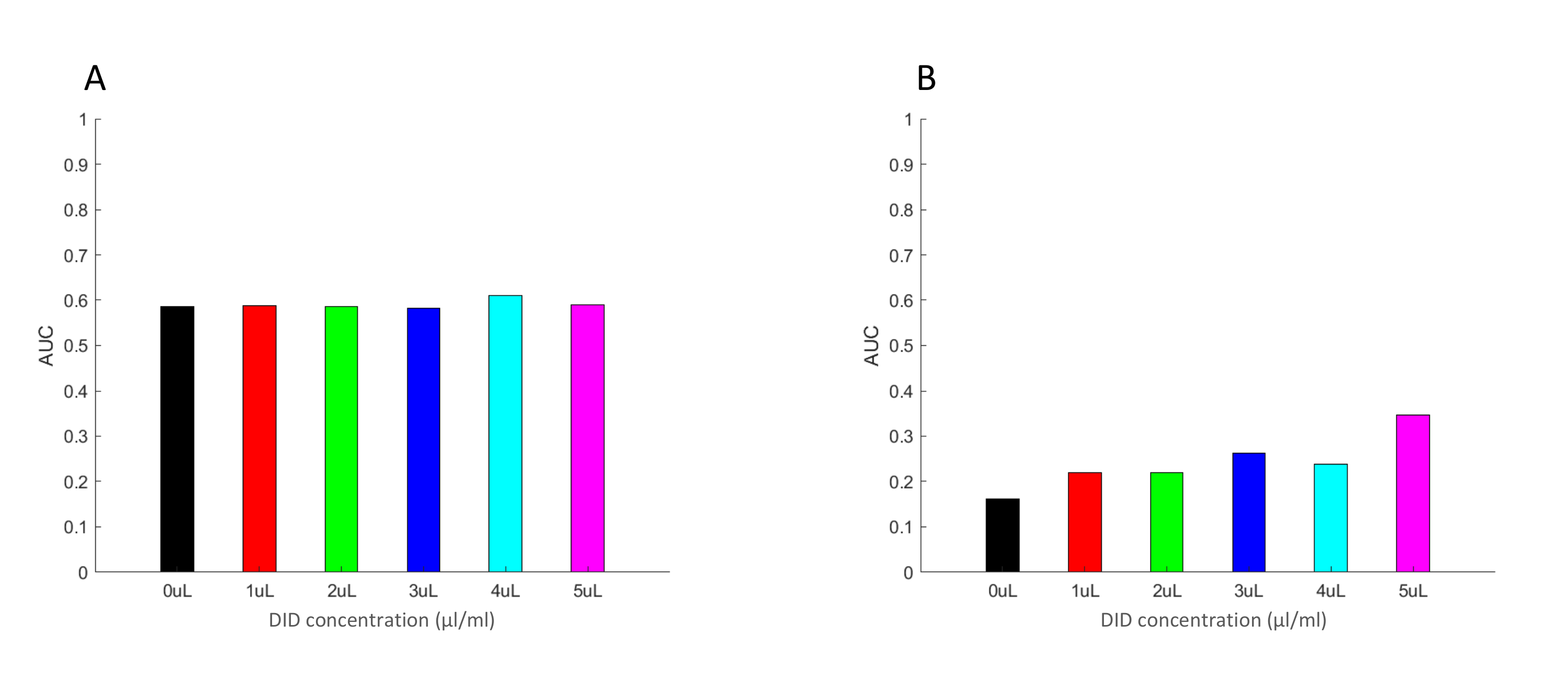

Supplement: FIGURE S15 — The area under the curve (AUC) of the PDF data from Supplementary Figure S11. (A) through (D): the AUC was calculated by multiplying the height of the individual columns by the width of the column in the histograms in panels (A) through (D) in Supplementary Figure S11, respectively. The zeroth columns (i.e., the MH-S macrophage cells without any adherent or phagocytosed spores) were left out of the integration. The FITC-free and FITC-labeled spores in (A) through (D) are labeled in black and red, accordingly. The AUC was calculated by using the equal weight of 1 for each column from Supplementary Figure S11, thus the AUC values mimic the symmetrized phagocytic index of the corresponding fungal species and staining conditons. The color scheme of the columns depicting the various DID concentrations between 0 and 5 μl/ml is the same as used in the legends of Supplementary Figure S11. [file Image_15.TIF]
